# WATER NEWS: a field approach for sustainable detection of pathogens in wastewater

**DOI:** 10.1093/nsr/nwaf275

**Published:** 2025-07-15

**Authors:** Zhou-Hua Cheng, Meng Du, Chen Qian, Shu-Xia Zhang, Hao-Da Wang, Wen-Wei Li, Dong-Feng Liu, Han-Qing Yu

**Affiliations:** State Key Laboratory of Advanced Environmental Technology, Department of Environmental Science and Engineering, University of Science and Technology of China, Hefei 230026, China; School of Life Sciences, University of Science and Technology of China, Hefei 230026, China; State Key Laboratory of Advanced Environmental Technology, Department of Environmental Science and Engineering, University of Science and Technology of China, Hefei 230026, China; State Key Laboratory of Advanced Environmental Technology, Department of Environmental Science and Engineering, University of Science and Technology of China, Hefei 230026, China; Fujian Institute of Hematology, Fujian Provincial Key Laboratory on Hematology, Fujian Medical University Union Hospital, Fuzhou 350001, China; State Key Laboratory of Advanced Environmental Technology, Department of Environmental Science and Engineering, University of Science and Technology of China, Hefei 230026, China; State Key Laboratory of Advanced Environmental Technology, Department of Environmental Science and Engineering, University of Science and Technology of China, Hefei 230026, China; School of Life Sciences, University of Science and Technology of China, Hefei 230026, China; State Key Laboratory of Advanced Environmental Technology, Department of Environmental Science and Engineering, University of Science and Technology of China, Hefei 230026, China; School of Life Sciences, University of Science and Technology of China, Hefei 230026, China; State Key Laboratory of Advanced Environmental Technology, Department of Environmental Science and Engineering, University of Science and Technology of China, Hefei 230026, China; School of Life Sciences, University of Science and Technology of China, Hefei 230026, China

**Keywords:** wastewater surveillance, field, CRISPR–diagnostic, pathogen detection, wastewater treatment plants

## Abstract

Wastewater surveillance is a critical tool in responding to the COVID-19 pandemic, yet traditional centralized methods are unsustainable due to their high costs and complex implementation requirements. Here, we introduce Wastewater Analysis and Tracking for Epidemiological Recognition: Necessary Early Warning System (WATER NEWS)—a field clustered regularly interspaced short palindromic repeats–diagnostic approach designed for robust and cost-effective pathogen detection in wastewater treatment plants (WWTPs). WATER NEWS combined a one-pot assay, an optimized reporter probe, on-site nucleic acid extraction, an improved freeze-drying process and a user-friendly, low-cost, battery-operated device, thereby overcoming the limitations of field-deployable wastewater surveillance. In evaluations across 25 WWTPs in 10 Chinese cities, WATER NEWS achieved sensitivities exceeding 90% and specificities of 100% in SARS-CoV-2 detection from the influent and effluent samples of WWTPs. Notably, the system consistently detected SARS-CoV-2 presence within 20 days at a local WWTP. Economic analysis reveals that our approach achieved a 6.5-fold cost reduction compared with clinical testing and costs nearly half that of traditional wastewater surveillance methods, making it a viable and sustainable option for wastewater surveillance.

## Teaser

A wastewater warning system -WATER NEWS is developed for the rapid field detection of SARS-CoV-2 and mpox at wastewater treatment plants.

## INTRODUCTION

Since the early twenty-first century, a series of infectious pathogens, including Middle East Respiratory Syndrome Coronavirus [1], Zika virus [2], Ebola virus [3], SARS-CoV-2 [4] and monkeypox virus (mpox) [5], have frequently emerged, leading to widespread infections and high mortality rates [6,7]. In this context, wastewater-based epidemiology (WBE) has increasingly demonstrated its value in pandemic monitoring and control [8–10]. Compared with clinical sample screening, wastewater surveillance shifts the focus from individual infections to community infection trends, providing a more efficient and comprehensive approach for epidemiological investigations [11,12]. Clinical diagnostics are frequently hampered by infected individuals’ avoiding testing or remaining undetected due to asymptomatic infection, alongside high costs, making sustained clinical testing economically burdensome and logistically challenging [13,14]. Conversely, wastewater surveillance provides insights into actual infection rates at a lower cost, establishing it as a sustainable tool for epidemiological surveillance [15–17].

Wastewater surveillance, as a cost-effective and noninvasive method, provides community-wide monitoring capabilities, offering crucial data support for health policy formulation [8]. It proved particularly valuable during the COVID-19 pandemic, in which analysing the concentration and viral genomic variations of SARS-CoV-2 ribonucleic acid (RNA) in wastewater effectively tracked transmission dynamics within catchment areas [18]. Additionally, correlating the concentration of SARS-CoV-2 RNA in wastewater with hospitalization rates helps to predict bed usage based on concentration fluctuations, enabling healthcare facilities to preemptively allocate medical resources [19]. Demonstrating recognition of its benefits, Hong Kong established 154 fixed monitoring stations covering 6 million people (∼80% of the total population) [20]. Meanwhile, the US Centers for Disease Control and Prevention developed a national wastewater surveillance system to support pandemic response efforts [21].

However, although dozens of countries and regions have implemented wastewater surveillance programs, only ∼16 low- and lower-middle-income countries and regions have adopted such programs due to the high costs and complex implementation requirements [22,23]. This represents only ∼23% of all countries and regions with such initiatives [8,24]. The field-deployable strategy based on clustered regularly interspaced short palindromic repeats–diagnostic (CRISPR–Dx) technology promises to reduce logistical and laboratory operation burdens, simplifying the monitoring process and potentially making it more suitable for areas lacking standard wastewater surveillance facilities [25–29]. Nonetheless, field application of this technology faces challenges such as lengthy processing times, nucleic acid aerosol contamination, temperature fluctuations and the inconsistent availability of refrigeration and power supply at field sites.

Here, we present a Cas12a-based one-pot wastewater warning system—WATER NEWS (Wastewater Analysis and Epidemiology Recognition: Necessary On-site Early Warning System)—enabling the rapid and efficient field detection of pathogens in wastewater. Using mpox and SARS-CoV-2 as representative pathogens, we developed a fast one-pot diagnostic method (15–20 min) by utilizing suboptimal adjacent motifs (PAMs) and optimized reporter probes to prevent nucleic acid aerosol contamination. Further, by optimizing the lyophilization process and designing compact diagnostic equipment, we eliminated the need for refrigeration and continuous electricity. The system (WATER NEWS) subsequently demonstrated high sensitivity and specificity during testing when the influent and effluent samples collected from 25 wastewater treatment plants (WWTPs) across 10 Chinese cities were used, supplemented by a 20-day field deployment at the Wangtang WWTP in Hefei, China. Through this work, a sustainable wastewater surveillance paradigm capable of detecting both DNA pathogens and RNA pathogens was successfully developed and applied (Table 1).

**Table 1.** Comparison of field CRISPR-based detection methods for human or wastewater samples.

	Detection	One/two	Time	Lyophilized	
Methods	method	steps	(min)	reagents	Samples
WATER NEWS (this work)	RT-RPA-Cas12a	One	15–20	Yes	Saliva/wastewater
SHERLOCK [26]	RT-RPA-T7-Cas13	Two	55	Yes	Saliva
DETECTR [35]	RPA-Cas12a	Two	45	No	Anal samples
sPAMC [38]	RT-RPA-Cas12a	One	15–20	No	Nasopharyngeal samples
SHINEv.1 [54]	RT-RPA-T7-Cas13	One	50	No	Nasopharyngeal samples
SHINEv.2 [40]	RT-RPA-T7-Cas13	One	90	Yes	Nasopharyngeal samples
Paper-Device [27]	RT-LAMP-Cas12a	Two	70	No	Wastewater

## RESULTS

### Design of a rapid one-pot detection method

**Figure 1.**
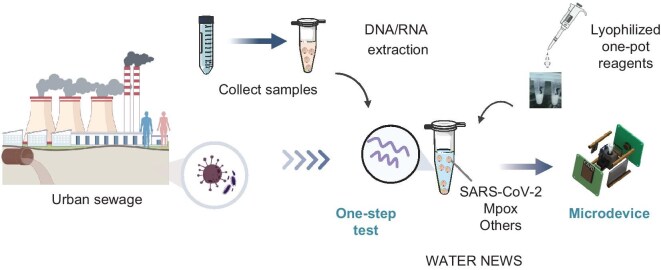
WATER NEWS for field wastewater surveillance. Enabling the concentration of pathogens, purification of nucleic acids, rapid one-pot detection and lyophilized reagents with the deployment of compact, battery-powered diagnostic microdevices. The schematics shown were created by figdraw.com.

**Figure 2.**
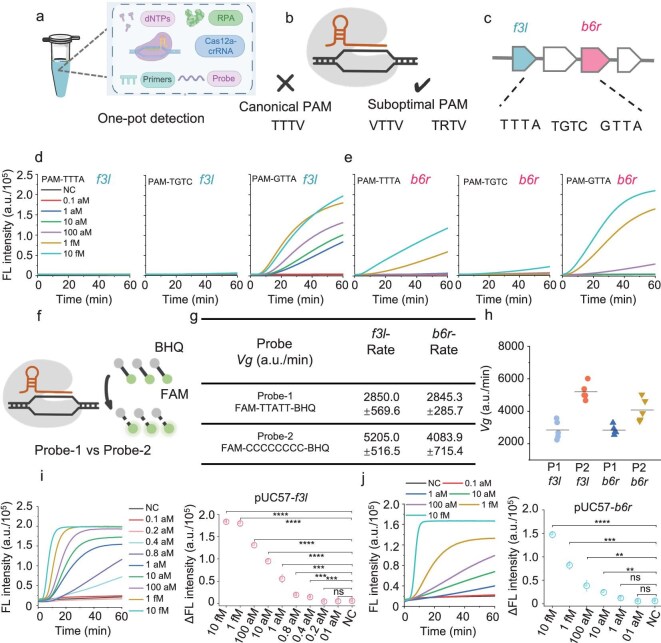
Design of one-pot assay for mpox detection. (a and b) Schematic illustrations of both canonical and suboptimal PAM-mediated *cis* cleavages in one-pot assay. (c) One canonical PAM (TTTA) and two suboptimal PAMs (TGTC, GTTA) were designed for the *f3l* and *b6r* genes. (d and e) The discrepancy performances in the one-pot test between canonical and suboptimal PAMs. Three crRNAs targeting one canonical PAM (TTTA) and two suboptimal PAMs were designed for the (d) *f3l* and (e) *b6r* genes, respectively. (f) Schematic illustrations of Probe-1- and Probe 2-mediated *trans* cleavages in one-pot assay. (g, h) Comparison of the cleavage rates of suboptimal PAM-mediated one-pot assays for *f3l* and *b6r* genes with two probes. (i and j) LoD of one-pot assays for the (i) *f3l* and (j) *b6r* genes using suboptimal PAM (GTTA) and Probe-2. The fluorescence values at 20 min of the one-pot reaction were compared after subtracting the initial fluorescence values. Mean ± SD of *n* = 3 technical replicates and NC represents reactions without substrate for (d and e) and (i and j). Statistical significance was analysed by using a two-tailed *t*-test: ns, *P* > 0.05, *P < 0.05, **P < 0.01, ***P < 0.001, ****P < 0.0001.

Field wastewater detection systems typically integrate nucleic acid preprocessing, amplification, automated detection and analysis, necessitating careful attention to detection efficiency, aerosol contamination prevention and reduced dependence on large-scale laboratory infrastructures and cold chain logistics (Fig. 1) [30]. To prevent nucleic acid aerosol contamination during the pipetting step, we selected the *f3l* and *b6r* genes from the mpox as targets to develop a one-pot detection method [31]. The *f3l* gene encodes the F3 protein, which suppresses the host cell's antiviral immune response and the *b6r* gene encodes an envelope protein; both serve as key detection targets for mpox [32,33]. Specifically, the recombinase polymerase amplification (RPA) reaction (37°C) and Cas12a-mediated detection occurred simultaneously in a single tube in the subsequent CRISPR assay (Fig. 2a) [34]. During the RPA process, a significant quantity of nucleic acid was amplified. The amplified product was then recognized and specifically cleaved by the Cas12a–crRNA complex, activating Cas12a's *trans*-cleavage activity. This activated nuclease subsequently cleaved fluorescent reporter probes, generating a fluorescence signal (Fig. S1a) [35]. Multiple crRNAs were designed to target the *f3l* and *b6r* genes with both canonical (TTTV) and suboptimal (e.g. TGTC, GTTA) PAMs for a one-pot reaction (Fig. 2b and c, and Fig. S1a and b). However, the detection sensitivity for trace nucleic acids when using canonical PAMs proved limited (Fig. 2d and e) [36,37]. In contrast, suboptimal PAMs, particularly GTTA, exhibited a faster reaction and higher sensitivity in the tests (Fig. 2d and e). The compatibility of the RPA and CRISPR components within the one-pot system may stem from the reduced *cis*-cleavage activity associated with the GTTA PAM, facilitating their simultaneous activity [38]. To rule out potential confounding effects from crRNA spacer sequence differences, we replaced the GTTA on the target sequence with TTTA and TGTC while using GTTA-targeting crRNA. Strong detection signals occurred exclusively with the GTTA target sequence (Fig. S2a and b).

To further improve the detection efficiency and shorten the detection time, we changed the sequences of the reporter molecules. We initially conducted a systematic screening experiment to evaluate the cleavage activity of Cas12a by using fluorescence reporter probes designed with different sequences (5A, 5G, 5C and 5T probes) under 50-nM *f3l* gene activator stimulation. The results reveal that the 5C probe achieved optimal *trans*-cleavage efficiency (Fig. S3a). To further optimize the detection system, we subsequently compared the performance between an extended 8C probe and the 5C probe. Notably, the 8C probe demonstrated superior cleavage kinetics (Fig. S3b) [39]. Subsequently, we evaluated the impact of fluorophore labeling on the performance of the 8C probe. The results demonstrate that probes labeled with either hexachloro-fluorescein (HEX) or 6-carboxy-fluorescein (FAM) exhibited similar fluorescence signal trends in one-pot detection assays, with no significant difference in the signal-to-noise ratios. Interestingly, under the same concentrations, the FAM-labeled probe yielded higher fluorescence intensity (Fig. S4a–c). Based on this finding, we designated the 8C probe with FAM as Probe-2 and systematically compared its detection efficiency with the existing Probe-1 (FAM–TTATT–BHQ) in the one-pot reaction system (Fig. 2f). The detection efficiencies of the two probe-mediated one-pot assays were compared by calculating the cleavage rates, defined as the fluorescence intensity at 20 min (after background subtraction) divided by the reaction time. Surprisingly, Probe-2 greatly improved the effectiveness of the one-pot detection and shortened the detection time to 15–20 min (Fig. 2g and h). The limit of detection (LoD) was as low as 0.4 aM (≈0.3 cp/μL) for *f3l* and 10 aM (≈6.0 cp/μL) for *b6r*, respectively (Fig. 2i and j). These results indicate that the selection of the appropriate suboptimal PAM and reporter probe was pivotal for the development of rapid one-pot nucleic acid detection approaches. Regarding probe selection, one-pot detection assays mediated by suboptimal PAMs revealed that cytosine-rich single-stranded DNA (ssDNA) probes significantly enhanced the detection efficiency. This improvement is likely due to the *trans*-cleavage activity of Cas12a, which, once activated, appears to preferentially target cytosine-rich ssDNA. Such a rapid isothermal trace nucleic acid detection method provides a cornerstone for field wastewater surveillance.

### Optimization of one-pot detection conditions for field diagnostic

We next explored the optimal output modality for the one-pot diagnostic method by comparing fluorescence and lateral-flow-strip assays for one-pot detection performance. To simulate the detection of actual virus samples, we applied a direct sample processing reagent (Sample Total Nucleic Acid Release Agent) to extract the *f3l* and *b6r* genes from adenovirus vector-based pseudoviruses. Initially, the copy numbers of pseudoviruses containing *f3l* or *b6r* genes were quantified via quantitative polymerase chain reaction (qPCR) by using plasmid standards (Fig. S5a and b). Then, we compared the release efficiency of *f3l* and *b6r* from these pseudoviruses by using lysis reagents (at 25°C for 1 min) and pyrolysis (at 95°C for 5 min). Compared with pyrolysis, the lysis reagent treatment in the one-pot assay resulted in a 10-fold lower LoD for *f3l* (3.9 cp/μL) and a 100-fold lower LoD for *b6r* (12.0 cp/μL) (Fig. S5c and e). The relatively low efficiency of the nucleic acid release by pyrolysis can be attributed to two key factors: (i) incomplete viral disruption, resulting in insufficient nucleic acid liberation; and (ii) co-precipitation and entrapment of nucleic acids during heat-induced protein denaturation [40]. In contrast, the commercially available direct nucleic acid release reagent employed in this study demonstrated superior performance. Its mechanism of action involves: (i) a synergistic effect between sodium hydroxide and surfactants in the reagent, which effectively disrupts lipid membranes and protein structures to facilitate the release of entrapped nucleic acids; and (ii) a strongly alkaline environment that not only markedly suppresses nuclease activity, but also promotes the dissociation of DNA-bound histones, thereby destabilizing the DNA double helix. These synergistic effects create optimal conditions for subsequent one-pot detection. Additionally, the LoD of the one-pot assay was also quantified by using a lateral-flow-based colorimetric readout. The LoD for *f3l* and *b6r* in this assay was 3.9 × 10^2^ and 1.2 × 10^3^ cp/μL, respectively (Fig. S5d and f). These findings indicate that the lateral-flow-strip assays were less sensitive compared with the assays based on fluorescent signaling.

**Figure 3.**
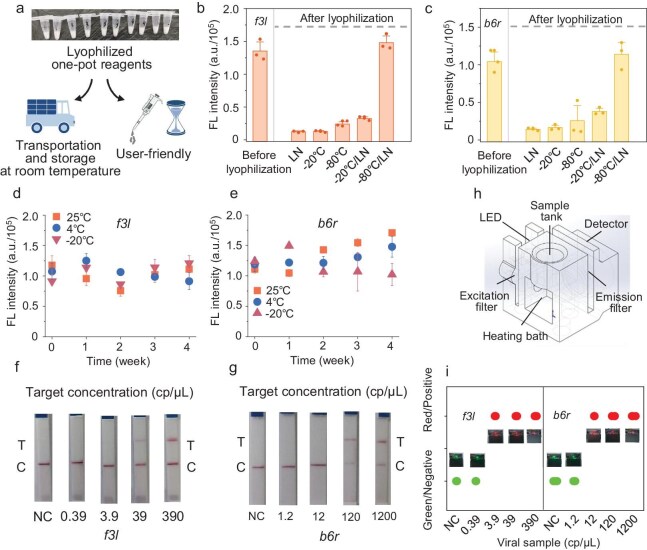
Optimizing lyophilization strategies for one-pot reactions. (a) Schematic of the benefits of lyophilizing one-pot reagents. (b and c) One-pot fluorescence on pseudoviruses (*f3l*–3.9 × 10^2^ and *b6r*–1.2 × 10^3^ cp/μl) before and after lyophilization by using the different freezing methods. ‘Before lyophilization’ refers to testing reagents that have not been freeze-dried. ‘LN’ denotes freeze-drying after initial freezing in liquid nitrogen, while ‘–20°C’ and ‘–80°C’ represent freeze-drying after being frozen at the respective temperatures. ‘–20°C/LN’ and ‘–80°C/LN’ indicate freezing for 2 h at –20°C or –80°C, respectively, followed by placement in liquid nitrogen for further freezing and lyophilization. Fluorescence was measured after 20 min. (d and e) One-pot fluorescence using lyophilized (–80°C/LN) reagents that were kept at 25°C, 4°C and –20°C. (f and g) Using hydrated reagents to determine the LoD of one-pot reaction with different concentrations of mpox DNA (f, *f3l* gene; g, *b6r* gene) based on chlorimetric readout. (h) Diagram of the portable microdevice. (i) Rapid detection of mpox with portable handheld reader. Virus loads: *f3l*: 0–3.9 × 10^2^ cp/μL, *b6r*: 0–1.2 × 10^3^ cp/μL. The schematics shown in (a) were created by figdraw.com.

Lyophilized reagents facilitate field CRISPR–Dx applications by reducing the dependency on refrigeration (Fig. 3a). However, a noticeable fluorescence drop occurred when one-pot reagents were instantly frozen in liquid nitrogen (Fig. 3b and c, and Fig. S6a and b). Other protective components such as 150 mM mannitol and 5% (w/v) sucrose failed to reactivate the one-pot assay (Fig. S6a and b) [40]. Additional freezing protocols were tested, including freezing at –20°C and –80°C (without pre-cooling). However, the resulting lyophilized powder produced weak fluorescence signals due to substantial physical loss during lyophilization (Fig. 3b and c). We then explored pre-freezing the test reagents at –20°C or –80°C for 2 h before flash-freezing in liquid nitrogen. Lyophilized reagents pre-cooled at –80°C exhibited full activity restoration (Fig. 3b and c). Meanwhile, the LoD of the one-pot assay with the dry powder was comparable to that with the original buffer (Fig. 2i and j, and Fig. S6c–f). To elucidate the rationale behind the effectiveness of this lyophilization strategy, we subjected the RPA components and the Cas12a–crRNA with fluorescent probe components to two distinct freezing protocols: direct freezing in liquid nitrogen and pre-cooling at –80°C for 2 h followed by liquid-nitrogen freezing. We then evaluated the post-lyophilization amplification efficiency of RPA and the *trans*-cleavage activity of Cas12a–crRNA. For RPA amplification testing, lyophilized powder obtained by both methods was used to amplify the *f3l* gene. After 20 min of amplification, Cas12a–crRNA and a fluorescent probe were added to assess the fluorescence activation. The results show that direct liquid-nitrogen freezing led to a significant reduction in the RPA amplification efficiency, whereas the pre-cooling strategy maintained robust amplification performance (Fig. S7a). This result suggests that rapid freezing might impair the activity of polymerase enzymes within the RPA mixture. To test this hypothesis, we supplemented the lyophilized RPA reagents (prepared via direct freezing) with fresh RPA polymerase, which restored the rapid amplification capacity (Fig. S7b). In contrast, for the Cas12a–crRNA and probe components, no significant difference was observed between the two lyophilization strategies (Fig. S7c). Together, these findings indicate that rapid freezing in liquid nitrogen compromised the enzyme stability in the RPA system, but had a minimal impact on the CRISPR detection components. The pre-cooling strategy effectively mitigated the detrimental impact of rapid liquid-nitrogen freezing on enzyme activity within the RPA system.

When calculating the average rate of fluorescence generation based on the difference between the fluorescence values at 20 min and the initial values, the optimized–lyophilized powder exhibited a fluorescence generation rate comparable to those of non-lyophilized controls (Fig. S8a–c). A 1-month stability study was conducted to assess the reagent stability at various temperatures over time. Lyophilized powders stored at 25°C, 4°C and –20°C for 1, 2, 3 and 4 weeks were rehydrated and tested by using lysis-treated pseudovirus samples. Activity was maintained for 4 weeks across all storage temperatures (Fig. 3d and e). Additionally, the lyophilized powder retained one-pot detection performance after being stored at 25°C for ∼50 days (Fig. S9a). To assess the impact of temperature fluctuations, the powder was sequentially stored at 10°C, 20°C and 30°C for 1 week each. Even after 3 weeks of variable temperature exposure, its detection performance remained robust (Fig. S9b). To assess whether lyophilization altered the lateral-flow-strip LoD, we repeated experiments using the freeze-dried one-pot assay. Results reveal that, while the LoD for lateral-flow detection increased by one order of magnitude post lyophilization, its sensitivity remained inferior to that of the fluorescence-based output (Fig. 3f and g). Additionally, lateral-flow strips can generate nucleic acid aerosol contamination during operation, making them poorly suited for field wastewater monitoring.

### Construction of integrated field diagnostic device

Given the single-use nature, insufficient sensitivity and the potential aerosol contamination of lateral-flow test strips, developing a reusable, compact and cost-effective device is crucial for facilitating field pathogen detection at wastewater sites [41]. Following the successful development of the freeze-dried one-pot diagnostic assay, we tried to fabricate an easy-to-use, low-cost, automated diagnostic device. Therefore, a Rubik's-Cube-sized device, composed of a temperature control component, a detection component, a control component and an outer casing, was fabricated by using a 3D-printing technique (Fig. 3h and Figs S10 and S11). The amplification chamber (operating at 37°C or 42°C) was equipped with light-emitting diodes and an optical filter for transillumination and fluorescent readout. After the nucleic acid sample was introduced into the collector of the amplification chamber containing hydrated one-pot buffer and the heater on the device was activated, the system automatically read fluorescence values every 5 seconds and compared them to a preset threshold. When the fluorescence exceeded the threshold for three consecutive readings, a red light signaled a positive result; otherwise, a green light indicated a negative result. Users can distinguish between positive and negative examples in as little as ∼20 min.

In addition, the sensitivity of this portable device was comparable to that of a plate-based fluorescence readout (Fig. 3i). The heating module and illumination system were battery-powered. To exhibit the detection process of the microdevice, a simple video was made to document its procedure for testing positive samples (Supplementary Information Video). Overall, this portable, real-time device facilitated the affordable, highly sensitive and accurate detection of pathogens in wastewater samples, overcoming the dependence on the expensive equipment that is prevalent in wastewater surveillance.

### Application of WATER NEWS for the detection of pathogens in wastewater samples

The specificity of the WATER NEWS was further validated by using five common pathogens (*Helicobacter pylori, Pseudomonas aeruginosa, Aeromonas hydrophila, Shewanella putrefaciens* and *Escherichia coli*) as potential interferents. A great amplification of fluorescence signals was observed only in the presence of pseudoviruses containing the *f3l* or *b6r* gene, without causing any cross-reactivity (Fig. S12). A crossover experiment was conducted to further confirm the high specificity of the WATER NEWS assay in the detection of mpox from unprocessed saliva (Fig. S13). We collected five saliva samples and divided them into different experimental groups. A portion of the saliva samples was mixed with pseudoviruses containing *f3l* or *b6r* as positive experimental groups, while the rest of the saliva samples without pseudoviruses were used as negative experimental groups. Viral loads in positive samples (*f3l*–3.9 × 10^2^ and *b6r*–1.2 × 10^3^ cp/μL) were comparable to the average in patients (cycle threshold (Ct) ≈ 28) [42]. WATER NEWS showed 100% sensitivity in the detection of mpox in all qPCR-positive samples. All samples identified as negative by using qPCR were also negative in our WATER NEWS assay (Fig. S13f and g). Additionally, considering the potentially low mpox load in actual positive samples, we introduced pseudoviruses at various concentrations into negative samples to simulate real scenarios. In saliva samples, the LoD of the WATER NEWS remained consistent with the previous findings (Fig. S14a and b). Moreover, the WATER NEWS successfully identified all positive samples, exhibiting Ct values between 28 and 34 (Fig. S14c and d).

**Figure 4.**
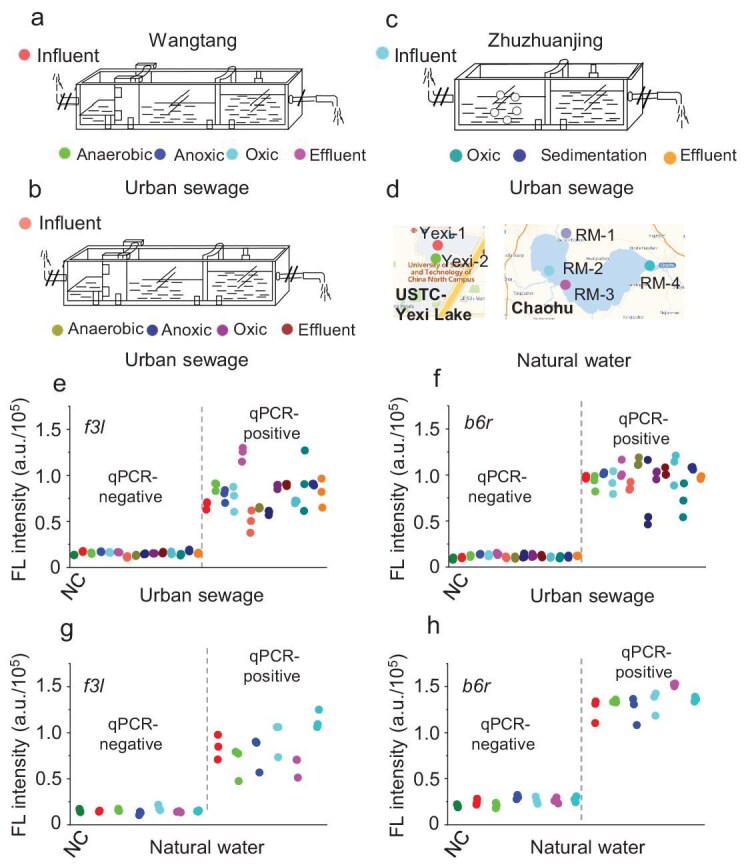
Mpox detection in municipal wastewater and natural water samples by WATER NEWS. (a–c) Wastewater samples were collected from the three WWTPs by adopting different treatment processes. (d) Natural water samples were collected from Yexi Lake and Chaohu Lake. (e and f) WATER NEWS assays of wastewater samples and contrived samples (e, *f3l* gene; f, *b6r* gene). The results were comparable to those of qPCR analysis. (g and h) WATER NEWS assays of the natural water samples and contrived samples (g, *f3l* gene; h, *b6r* gene). The results were comparable to those of qPCR analysis. Virus loads: *f3l*–3.9 × 10^2^ and *b6r*–1.2 × 10^3^ cp/μL.

To evaluate the feasibility for mpox testing in wastewater with WATER NEWS, we collected samples across the different treatment units of the three WWTPs in Hefei City, China, on 14 July 2022 and tested them with WATER NEWS (Fig. S15). Samples from Zhuzhuanjing WWTP included the influent, aeration tank, sedimentation tank and effluent; samples from Wangtang and Hudaying WWTPs included the influent, anoxic tank, anaerobic tank, oxic tank and effluent (Fig. 4a–c). Concentrated and extracted nucleic acids from these wastewater samples were analysed by using WATER NEWS. Meanwhile, pseudoviruses were also mixed with some samples as positive controls. Field testing confirmed the absence of mpox in wastewater samples across all three WWTPs on 14 July 2022, while all the contrived samples showed positive signals (Fig. 4e and f, and Figs S16–S18 and S20). We further tested surface water samples from Yexi Lake (University of Science and Technology of China campus) and Chaohu Lake—one of China's largest freshwater lakes (Fig. 4d). Similarly, no mpox was detected in these natural water samples, but WATER NEWS correctly identified the presence of mpox in all the contrived water samples, demonstrating 100% concordance with qPCR (Fig. 4g and h, and Figs S19 and S20). Collectively, these results confirm the high reliability and broad applicability of the WATER NEWS for mpox surveillance.

To further validate the versatility of the WATER NEWS platform, we applied it for the detection of both bacterial and fungal targets in wastewater by using *P. aeruginosa* and *Candida albicans* as representative organisms. For *P. aeruginosa*, we established a one-pot detection assay targeting the *miaA* gene. The results demonstrate that the LoD of WATER NEWS was comparable to that of qPCR, reaching as low as 1 aM (Fig. S21a–c). We then applied the assay to influent samples collected from 24 WWTPs across 9 cities in China (Fig. S21d). The majority of the samples tested positive for *P. aeruginosa*, with the WATER NEWS assay achieving a sensitivity of 95.2% and a specificity of 100% (Fig. S21e and f). Similarly, for *C. albicans*, the CHS1 gene was selected as the detection target. The assay again exhibited high sensitivity and specificity. However, unlike *P. aeruginosa, C. albicans* was not detected in most of the influent samples (Fig. S21a–f).

**Figure 5.**
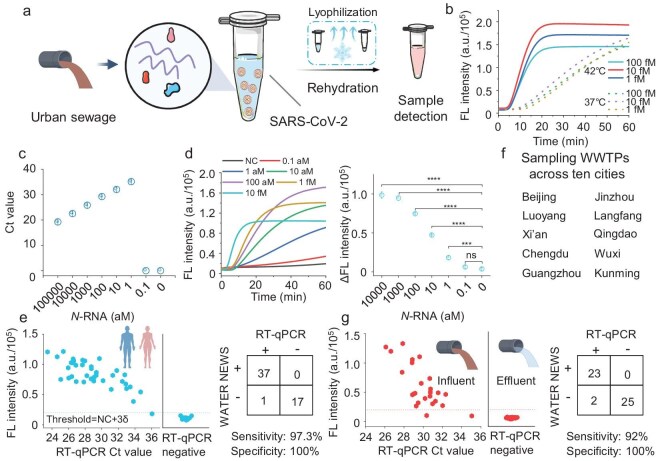
SARS-COV-2 detection in human saliva and urban sewage by WATER NEWS. (a) Schematic diagram illustrating the detection of SARS-COV-2 in saliva and sewage. (b) Comparison of the detection efficiency of WATER NEWS for *N* gene with 1, 10 or 100 fM at 37°C and 42°C. (c and d) Comparison of the detection limit of *N* gene by using RT–qPCR and WATER NEWS. The fluorescence values at 20 min of the one-pot reaction were compared after subtracting the initial fluorescence values. (e) Using WATER NEWS to detect both positive and negative samples from human saliva. (f and g) Using WATER NEWS to detect the influent and effluent samples from 25 WWTPs in 10 cities of China. Sensitivity: the proportion of true positives correctly identified by the test. Specificity: the proportion of true negatives correctly identified by the test. The threshold was set as the mean of three negative experiments plus three times the standard deviation. If the fluorescence value exceeded this threshold, then it was defined as positive; otherwise, it was defined as negative. Statistical significance was analysed by using a two-tailed *t*-test: ns, *P* > 0.05, *P < 0.05, **P < 0.01, ***P < 0.001, ****P < 0.0001. The schematics shown in (a, e, g) were created by figdraw.com.

Beyond DNA pathogens, we also explored the applicability of the one-pot detection methods for RNA pathogens. Using SARS-CoV-2 as a case study, we designed a crRNA targeting the *N* gene for one-pot analysis (Fig. 5a). However, the detection efficiency for SARS-CoV-2 decreased at 37°C compared with mpox detection, possibly due to the inefficiency of reverse transcriptase in transcribing RNA to complementary DNA​ (cDNA) at this temperature [38]. A higher detection efficiency for SARS-CoV-2 was achieved when the temperature was set at 42°C (Fig. 5b). Furthermore, WATER NEWS demonstrated sensitivity that was comparable to that of reverse transcription quantitative polymerase chain reaction (RT-qPCR), detecting SARS-CoV-2 RNA at concentrations as low as 1 aM within 20 min (Fig. 5c and d). Consistently with the mpox specificity results, the system generated no detectable fluorescence when challenged with non-target pathogens, confirming the target specificity (Fig. S23).

Subsequently, we assessed WATER NEWS performance when using 55 clinical saliva samples, comparing the results against RT–qPCR. WATER NEWS correctly identified 37 of 38 positive patient samples and all 17 negative samples (Fig. 5e). In addition, we evaluated the performance of WATER NEWS when using influent and effluent samples from 25 WWTPs across 10 cities in China (including one additional WWTP from Wuxi city) (Fig. 5f). The results were compared with those obtained by using RT–qPCR to assess its effectiveness in local epidemic surveillance. The results show that WATER NEWS had a sensitivity of >90% and a specificity of 100% when compared with RT–qPCR (Fig. 5g). Notably, a significant amount of SARS-CoV-2 was detected in the influent samples of all WWTPs, but not in the effluent samples of the secondary sedimentation tank, indicating effective viral removal during treatment. This observation is consistent with our previous work. In the course of long‑term wastewater surveillance, we found that, during periods of low SARS‑CoV‑2 transmission, viral RNA in the treated effluent was virtually undetectable [43]. This result suggests that the activated sludge might play a pivotal role in virus removal from municipal wastewater [44,45]. Thus, appropriate treatment of excess sludge might effectively prevent the spreading of pathogenic viruses from the urban population to the natural environment [46]. As the COVID-19 pandemic gradually subsided, the same set of 25 WWTP influent samples was collected again on 10 January 2023 (Fig. S24a). Despite the lower concentration of SARS-CoV-2 in these samples, WATER NEWS could successfully identify 23 out of 24 positive samples and the sole negative sample was also accurately detected (Fig. S24b and c). These results demonstrate that WATER NEWS has great potential for field monitoring at wastewater sites.

### Implementation of WATER NEWS in WWTPs

**Figure 6.**
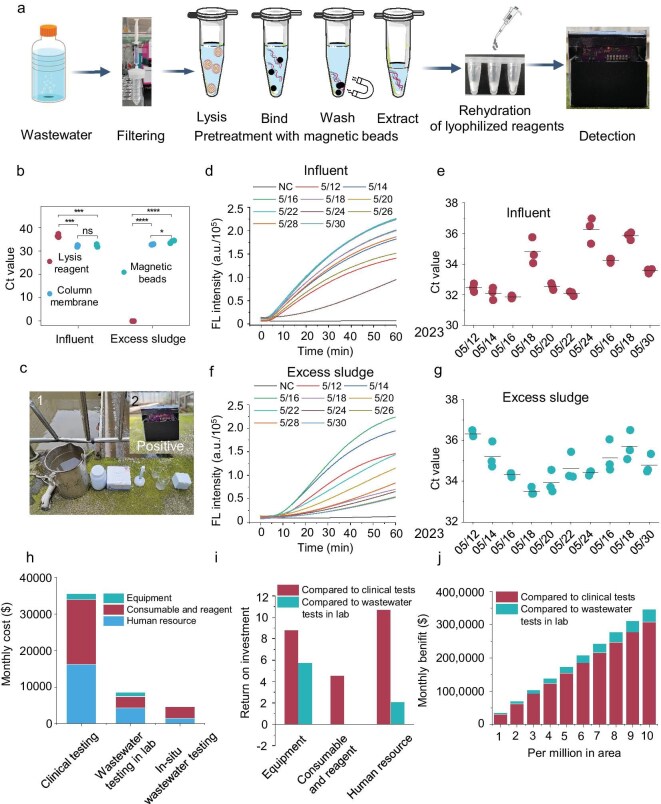
Real-time and field early warning of COVID-19 in Hefei City by using WATER NEWS. (a) Schematic diagram illustrating the field deployment for diagnosing SARS-CoV-2 in wastewater. (b) Comparison of the efficiency of magnetic bead adsorption and column membrane method for capturing SARS-CoV-2 nucleic acids in influent and excess sludge. (c) Real-time and field detection of SARS-CoV-2 in influent and excess sludge from WWTPs with on-site images. (d and f) Re-examination of the field diagnostic results by using WATER NEWS in the laboratory. (e and g) Confirmation of WATER NEWS results by using RT–qPCR. (h) Monthly costs of clinical testing, standard wastewater surveillance paradigm and WATER NEWS. (i) Comparison of the costs and benefits using the ROI. (j) Monthly cost savings (benefits) of WATER NEWS based on different monitored population sizes. Statistical significance was analysed by using a two-tailed *t*-test: ns, *P* > 0.05, *P < 0.05, **P < 0.01, ***P < 0.001, ****P < 0.0001.

Compared with traditional wastewater surveillance paradigms, which involve collecting samples from sampling points and transporting them to centralized laboratories for testing, our WATER NEWS has the potential for rapid field diagnosis at wastewater sites. This capability significantly reduces economic costs and labor requirements. To validate its monitoring capacity, we conducted 10 consecutive field tests at Hefei's Wangtang WWTP over 20 days (every 2 days). Initially, to enhance the sensitivity of WATER NEWS and further improve its application for field diagnosis in wastewater, we integrated WATER NEWS with magnetic bead adsorption technology. The latter's superior nucleic acid purification ability enhanced the efficiency of nucleic acid extraction from wastewater (Fig. 6a) [18]. Results show that the nucleic acid yield obtained through magnetic bead adsorption was comparable to that obtained by using the column membrane purification method in the laboratory (Fig. 6b). Subsequent field monitoring results indicated the presence of SARS-CoV-2 in all 10 consecutive tests conducted during the second wave of COVID-19 infections that began in Hefei in May 2023 (Fig. 6c). Field results were validated by using both WATER NEWS and RT–qPCR in the laboratory (Fig. 6d–g). Notably, WATER NEWS consistently detected viral signals despite inherent wastewater concentration fluctuations (Fig. 6d and e). As the Ct value of the sample approximated 36, the fluorescence intensity tended to wane. Meanwhile, the quantitative results from RT–qPCR also indicated that the second wave of the epidemic was waning in Hefei City (Fig. 6f and g).

We further employed a micro-costing method to compare the costs of WATER NEWS against clinical testing and traditional wastewater surveillance paradigms [47,48]. Cost calculations were based on a hypothetical population of 1 million, with 100–1000 infections per day [49,50]. Detailed comparisons were made of the equipment, consumables, reagents and human resource costs required for clinical sampling, wastewater sampling, transportation and nucleic acid testing (Tables S8–S10). Results indicate that, even at 100 infections/day/million, clinical testing incurred significantly higher costs than wastewater surveillance (Fig. 6h). WATER NEWS achieved a 6.5-fold cost reduction versus clinical testing and approximately halved the costs relative to standard wastewater surveillance. Primary savings were attributed to equipment and human resources, with return on investment (ROI) values of 5.8 and 2.1 when compared with standard wastewater monitoring paradigms (Fig. 6i). Cost advantages amplified as the population monitored was increased from 1 to 10 million (Fig. 6j). It should be noted that our economic analysis did not consider the costs and benefits of a hybrid model that partially employs wastewater surveillance and clinical testing. Further work is needed to provide a more accurate economic analysis by utilizing various hybrid models. Collectively, these results demonstrate that WATER NEWS can exhibit effective and stable wastewater monitoring at a lower cost, making it a viable and sustainable option for low- and lower-middle-income countries and remote rural areas.

## DISCUSSION

The continued development of WBE calls for field wastewater surveillance methods [25,28]. To meet the great challenges during testing at wastewater sites, preferable technology should meet the following standards: rapid response, no risk of nucleic acid aerosol contamination, high sensitivity and specificity, and deployability in the field. High sensitivity and specificity are particularly critical for wastewater surveillance, especially in the post-pandemic era, when SARS-CoV-2 is typically present at very low levels in sewage [43]. Moreover, monitoring low-abundance viral signals in wastewater from transportation hubs or airports—key points of interregional human mobility—will be essential for advancing WBE [51]. To this end, we have developed WATER NEWS—a high-sensitivity and high-specificity nucleic acid detection method (Table 1). This method offers significant advantages for wastewater monitoring in several ways: (i) WATER NEWS eliminates the nucleic acid aerosol contamination issue present in traditional two-step methods (Fig. 2d and e); (ii) the speed of WATER NEWS is fast, requiring only 15–20 min, which is two to three times faster than the traditional CRISPR–Dx methods (Fig. 2i and j); (iii) WATER NEWS demonstrates high sensitivity and specificity when processing both saliva samples and complex wastewater samples (Fig. 5e and g); and (iv) the use of lyophilized reagents and a rechargeable microdevice frees WATER NEWS from the need for refrigeration and power supply (Figs 3d and e, and 6c).

The above features enable WATER NEWS to provide sustainable and effective monitoring at wastewater sites. Compared with traditional wastewater surveillance methods, WATER NEWS offers enhanced field deployability and low-cost operation, facilitating deployment at diverse sampling points—such as residential areas, schools, hospitals and transportation hubs—to strengthen early outbreak detection. More importantly, its low-cost characteristic makes it particularly suitable for economically underdeveloped and remote areas, which face heightened risks of emerging ‘X pathogen’ threats due to limited healthcare infrastructures [8]. Therefore, establishing a low-cost, high-sensitivity and high-specificity field wastewater monitoring system will provide sustainable health support for populations in these regions.

In addition, public concern about health risks now extends to pathogens beyond SARS-CoV-2 that are capable of causing localized outbreaks, such as influenza virus and *Mycoplasma pneumoniae*. WATER NEWS has demonstrated high applicability for both DNA pathogens (e.g. mpox, *P. aeruginosa* and *C. albicans*) and RNA pathogens (e.g. SARS-CoV-2). Therefore, by simply replacing the RPA primers and crRNA recognition sequences, WATER NEWS can be easily expanded to detect other pathogens, offering broad applicability. This versatility enables WATER NEWS to quickly adapt to and identify newly emerging or mutating pathogens, greatly enhancing its value in wastewater surveillance.

In summary, WATER NEWS appears to be the first field wastewater surveillance method to exhibit the combination of one-pot use, great sensitivity and specificity, high speed and broad applicability. Compared with our prior CRISPR–Dx-based detection of antibiotic resistance genes in urban water cycles (which remains confined to laboratory-based environmental sample analysis) [34], this study overcomes critical challenges including the optimization of lyophilization processes and compact instrument design. By advancing the one-pot RNA detection methodology, we have achieved field-deployable SARS-CoV-2 monitoring in real-world environmental settings. Despite these advancements, there is still a need to develop field nucleic acid diagnostic techniques that can perform semi-quantitative or quantitative analyses for wastewater surveillance. For instance, by selecting different PAM sequences, one can establish LoD gradients at attomolar (aM), femtomolar (fM) and picomolar (pM) levels to enable semi-quantitative measurement. Alternatively, the targeted mutagenesis of the Cas12 protein and the repeat regions within crRNA can yield a series of Cas12a–crRNA complexes with distinct cleavage activities, which can then be assembled into an LoD-gradient array for semi-quantitative detection. Meanwhile, multiple pathogens may coexist, necessitating the upgrade of portable devices to enable simultaneous multichannel testing. In addition, simplifying the nucleic acid extraction step remains one of the key bottlenecks limiting the on-site monitoring of wastewater. Integrating rapid nucleic acid extraction with paper-based materials may offer a promising solution [52]. Most crucially, we believe that the field monitoring strategy, when implemented at various wastewater sites in future practical engineering operations, will provide timely field monitoring data, facilitating the establishment of a reliable and sustainable epidemic warning system.

## CONCLUSION

In summary, we have developed a stable, cost-effective pathogen wastewater surveillance strategy for field deployment. Our one-pot detection design mitigates nucleic acid aerosol contamination risks. Optimizing lyophilization eliminates the reliance on cold chain logistics for transport and storage. Furthermore, the introduction of a rechargeable miniaturized diagnostic device has enhanced the stability and applicability of the monitoring approach. This study provides a supplementary option for exploring sustainable wastewater monitoring models in the post-pandemic era, with the potential to substantially reduce the economic and labor costs associated with wastewater surveillance.

## METHODS

### Plasmid construction, pseudovirus and RNA standards preparation

The *f3l* and *b6r* genes of mpox_USA_2022_MA001 (accession: ON563414), the *miaA* gene of *P. aeruginosa*, the *CHS 1* gene of *C. albicans* and the *N* gene of SARS-CoV-2 (accession: MN975262.1) with T7 promoter were synthesized (General Bio Co., China) and cloned into the pUC57 vector. The monkeypox pseudovirus was an AD5 replication-deficient adenovirus packaged with the mpox *f3l* or *b6r* gene (Sangon Biotech Co., China). The mpox samples were prepared by diluting the plasmids or pseudovirus at a ratio of 1:100 in lysis buffer (Sample Total Nucleic Acid Release Agent, Genestonebio Co., China). SARS-CoV-2 *N*-gene transcripts were obtained through transcription and gradually diluted to standard concentrations. A standard curve was generated by using RT–qPCR. Viral loads were quantified by comparing them with a standard curve generated by using plasmid DNA in qPCR.

### qPCR and RT–qPCR assay*s*

qPCR assays for plasmid standards and samples containing mpox were performed in 20-μL reaction volumes containing 10 μL 2 × AceQ qPCR probe master mix (Nanjing Vazyme Biotech Co., China), 1 μL of each primer pair at 10 μM and 0.2 μL 10 μM TaqMan probe (Sangon Biotech Co., China). RT–qPCR assays for SARS-CoV-2 samples in 20-μL reaction volumes contained 10 μL 2 × AceQ qPCR probe master mix, 0.5 μL reverse transcriptase (Shanghai Takara Bio Co., China) and 1 μL of each primer pair at 10 μM and 0.2 μL 10 μM TaqMan probe. The primers and TaqMan probes are listed in Table S3. The Minimum Information for Publication of Quantitative Real-Time PCR Experiments checklist is given in Table S4 [53].

### One-pot assay

The comparison of conventional and suboptimal PAMs in the one-pot assay was performed by using varying concentrations of plasmid standards. The lyophilized RPA pellet (Weifang Amp-Future Biotech Co., China) was reconstituted in a reaction mixture comprising 29.4 μL of buffer A, 2 μL each of 20 μM RPA forward and reverse primers, 400 nM FQ-ssDNA reporter (Probe-1 or Probe-2), 100 nM LbCas12a, 200 nM crRNA and nuclease-free water. Upon adding 3 μL of buffer B to such a 24-μL mixture, a 3-μL sample was incubated at 37°C. The fluorescence was measured by using a SpectraMax i3x Multi-Mode Microplate Reader (excitation at 485 nm and emission at 520 nm). For lateral-flow-strip detection, a FAM and biotin-labeled Probe-2 replaced the FQ-labeled Probe-2 at a final concentration of 50 nM. SARS-CoV-2 *N*-gene transcripts were obtained through transcription and gradually diluted to standard concentrations. To evaluate the LoD of RNA and assay performance on real samples, 0.8 μL of reverse transcriptase (Shanghai Takara Bio Co., China) was added to the one-pot reaction mixture, followed by fluorescence measurements at 37°C or 42°C. Primer and probe sequences are detailed in Table S3.

## Supplementary Material

nwaf275_Supplemental_Files

## Data Availability

Data obtained and used during the current work are provided in the Supplementary information. The strains and plasmids are available upon request.
