# Supplementary material for: WATER NEWS: a field approach for sustainable detection of pathogens in wastewater
Source: Natl Sci Rev. 2025 Jul 15;12(8):nwaf275. doi: 10.1093/nsr/nwaf275 (PMC12365751; doi:10.1093/nsr/nwaf275)
Supplement: nwaf275_Supplemental_Files [file nwaf275_supplemental_files.zip › NSR_MS-2025-170.R1-SI-2025-7-1-ok.docx]

***Supplementary Information for***

**WATER NEWS: A Field Approach for Sustainable Detection of Pathogens in Wastewater**

Zhou-Hua Cheng^1,2^, Meng Du^1^, Chen Qian^1^, Shu-Xia Zhang^3^, Hao-Da Wang^1^, Wen-Wei Li^1,2^, Dong-Feng Liu^1,2,*^, Han-Qing Yu^1,2,*^

^1^State Key Laboratory of Advanced Environmental Technology,

Department of Environmental Science and Engineering, University of Science and Technology of China, 230026, Hefei, China

^2^School of Life Sciences, University of Science and Technology of China, 230026, Hefei, China

^3^Fujian Institute of Hematology, Fujian Provincial Key Laboratory on Hematology, Fujian Medical University Union Hospital, 350001, Fuzhou, China

This PDF includes supplementary tables 1-10, supplementary figures 1-24 and references.

**METHODS**

**Expression, and purification of LbCas12a protein**

pET28a-LbCas12a, an overexpression plasmid, was transformed into *Escherichia coli* BL21 Rosetta. For protein expression, individual clones were grown overnight in 5 mL Luria-Bertani (LB) and then transferred 3 mL cultures into 3 L fresh LB medium. The strains were grown at 37 °C and 220 rpm. Isopropyl β-D-1-thiogalactopyranoside was added as an inducer at a final concentration of 0.2 mM until the value of OD600 reached 0.8 and incubated at 16 °C for an additional 16 h. Cells were harvested by centrifugation and resuspended in the buffer (50 mM tris-HCl (pH 8.0) and 1.5 M NaCl) for sonication. The resultant supernatant underwent further Ni-NTA agarose chromatography purification. Using the modified Bradford Protein Assay Dye Kit based on bovine serum albumin, the concentration of the purified LbCas12a was measured. The LbCas12a was stored at −80 °C for later use.

**Preparation of crRNA**

To prepare the transcription template for crRNA, two oligos were heated to 95 °C for 4 min and then cooled at a rate of 0.1 °C/s to 4 °C. The T7 promoter sequence was present in an oligo, whereas the handle and spacer sequences were present in another. The transcription template was incubated with T7 RNA polymerase for 2 h at 37 °C to synthesize the target crRNA. The transcription product was purified by digesting the DNA template using DNase I at 37 °C for 15 min and removing impurities using an RNA Purification Concentration Kit (Jianshi Biotechnology Co., China). The crRNA and target DNA sequences with different PAMs are presented in the Supplementary Tables 1-2.

**Comparison of the lysis efficiency with the lysis reagents and pyrolysis**

Pseudoviruses carrying the *f3l* or *b6r* gene were diluted at a ratio of 1:100 in lysis buffer (Sample Total Nucleic Acid Release Agent, Genestonebio Co., China) at room temperature and shaken for 1 min. Meanwhile, pseudoviruses were also diluted with nuclease-free water and incubated at 95 °C for 5 min. Then, these samples were detected by one-pot assay, and the fluorescence value was measured after the reaction was conducted at 37 °C for 20 min.

**Lyophilisation optimization for one-pot assay**

To obtain the lyophilized powder, 27 µL of reaction reagent—consisting of 24 µL of one-tube reaction mixture and 3 µL of Buffer B—was first frozen at -20 °C or -80 °C for 2 h (ensuring that the reagent was completely frozen), followed by rapid freezing in liquid nitrogen, and finally lyophilized at 5 Pa using a vacuum freeze dryer for 3 h. It is important to note that the microtubes remained sealed throughout the freezing process. After liquid nitrogen freezing, the caps were opened and the tube openings were covered with filter paper during the drying step. Meanwhile, the reaction reagent was also lyophilized at 5 Pa using a vacuum freeze dryer for 3 h after being frozen at -80 °C, -20 °C, and liquid nitrogen, respectively. The impact of dosing 5% sucrose or 150 mM mannitol into reaction mixture on lyophilisation was also examined as described above. When required, lyophilized powder was rehydrated with 27 µL nuclease-free water. The stability of lyophilized powder was tested with the one-pot assay after lyophilized powder was kept at room temperature, 4 °C, or -20 °C for 7, 14, 21, and 28 d. Additionally, the lyophilized powder was stored at room temperature for 50 days and subsequently evaluated for its one-pot detection performance targeting 1 fM *f3l* gene. To assess the impact of temperature fluctuations on reagent stability, the powder was sequentially stored at 10 °C, 20 °C, and 30 °C for one week each, followed by performance testing of the one-pot assay.

**Sample collection**

For the specificity of the WATER NEWS assay, *Helicobacter pylori*, *Pseudomonas aeruginosa* PAO1, *Aeromonas hydrophila* ATCC 7966, *Shewanella putrefaciens* CN32, and *Escherichia coli* MG1655 were employed as interfering samples. *Helicobacter pylori* was purchased from the Guangdong Microbial Culture Collection Center (GDMCC), China, with the accession number GDMCC 1.2654. The wastewater samples were collected from the different treatment units of the three WWTPs (Wangtang, Hudaying, and Zhuzhuanjing) in Hefei City, China, on July 14, 2022. The natural water samples were collected from Chaohu and Yexi Lake in Hefei City, China, on May 15, 2022. Similarly, the wastewater and natural water samples with pseudoviruses acted as positive groups. The information about the sampling locations in Chaohu and Yexi Lake is given in the Supplementary Table 5.

The clinical samples tested in this work was approved by the Ethics Committee of the Fujian Medical University Union Hospital (2024KY093). The saliva samples were collected from the Fujian Medical University Union Hospital that were also confirmed by RT-qPCR. Samples of the influents and secondary sedimentation tank effluents were collected from 25 WWTPs located in 10 cities across China on December 26, 2022 and January 10, 2023. The selected WWTPs vary in their processing techniques, chosen to represent different cities, and also differ significantly in their treatment capacities. Detailed information is available in the Supplementary Tables 6-7. After collection, wastewater samples were stored at 0 to 4 °C and transported to the laboratory for analysis within 48 h.

**Comparison of the nucleic acid yield between magnetic bead adsorption and columnar membrane retention method**

Wastewater samples (50 mL each) were first filtered through a 0.22 µm aqueous filter to remove larger particles. Next, 12 mL of a PEG mixture (comprising 50% PEG and 10% NaCl, w/v) was added [1]. The solution was then passed through another 0.22 µm filter to concentrate the viruses. For nucleic acid extraction, a magnetic bead adsorption technique was used. This involved cleaning and lysing the viruses on the filter with a lysis reagent compatible with magnetic beads. Prepared magnetic beads were added, thoroughly mixed, and placed on a magnetic rack. After removing the supernatant, the beads were washed first with the provided washing liquid and then with 75% ethanol. Post ethanol evaporation, 50 µL of DEPC water was added to elute the nucleic acids from the beads. Additionally, a columnar membrane retention method using a total RNA extraction and purification kit (Karroten Biotech Co., China) was employed. For sludge samples, 0.1 g of sludge was mixed with 2 mL lysis reagent and homogenized. The homogenate was then filtered through a 0.45 µm filter to remove larger particles, followed by the addition of magnetic beads to the filtrate for nucleic acid enrichment. For all the aforementioned samples, A lentivirus possessing structural and biological characteristics akin to SARS-CoV-2, carrying the green fluorescent protein biomarker gene, was introduced as a recovery control (Sangon Biotech Co., China). Negative control (water) and positive control (the lentivirus containing the SARS-CoV-2 *N* gene, Sangon Biotech Co., China) were processed using the same extraction protocol. If the recovery rate of the positive control dropped below 10% or the negative control exhibited a positive signal, the extraction procedure was repeated. Each sample underwent duplicate nucleic acid extractions, and the resulting nucleic acids were tested in triplicate via RT-qPCR and WATER NEWS.

**WATER NEWS testing process**

Nucleic acid samples (3 µL) were combined with a 27 µL reaction mixture in a tube. The fluorescence in the WATER NEWS assay was monitored using a SpectraMax i3x Multi-Mode Microplate Reader. For the lateral flow readout in WATER NEWS, 20 µL of nuclease-free water was added to the mixture containing 3 µL nucleic acids 30 min post initiation of the assay. Subsequently, a lateral flow strip was immersed in the tube, and 2 min later, it was photographed for analysis. Additionally, WATER NEWS was evaluated using the microdevice. In this process, 3 µL of nucleic acid samples was introduced into a collector tube containing 27 µL reaction mixture within the amplification chamber. The device then heated the tube to 37-38 °C (or ~42 °C for SARS-CoV-2), measured background fluorescence, and continuously read the fluorescence every 5 s after subtracting the background value. The device emitted green or red light if the fluorescence was below or above the preset threshold, respectively.

**Establishment of the field warning system**

Wangtang WWTP in Hefei City serves as the subject for the field detection of the SARS-CoV-2. A magnetic bead reagent kit, freeze-dried dry powder for one-pot assay, and a portable microdevice were transported to the Wangtang WWTP. Employing the methodologies aforementioned, field wastewaters and excess sludge were tested for the presence of the SARS-CoV-2. Samples obtained via magnetic bead adsorption were directly employed with lyophilized one-pot powder and then detected with the microdevice for SARS-CoV-2 diagnostics.

**Economic and cost analysis**

The micro-costing method was used to detail the resources and costs associated with nucleic acid testing based on clinical samples, wastewater samples testing in lab, and field wastewater monitoring. The sum of financial and opportunity costs provided an estimate of the economic cost [2, 3]. The cost calculation included equipment costs, consumables and reagent costs, and labor or human resources costs. Equipment costs were estimated on a monthly basis, considering the assumed lifespan and the proportion allocated for SARS-CoV-2 usage (Supplementary Table 8). Consumables and reagent costs were calculated based on the estimated monthly sample volume (Supplementary Table 9). Labor and human resource costs covered both full-time and part-time personnel involved in SARS-CoV-2 testing activities, with costs calculated based on their time spent on these activities (Supplementary Table 10). All expenses were reported in 2024 US dollars ($). The exchange rate used for conversion from Chinese Yuan (RMB) was 0.138 US dollars per RMB. The key metric estimated in this cost study was the total monthly cost and the return on investment (ROI) [4].

ROI = Benefit/Cost

The benefit was defined as the saving cost of field wastewater monitoring compared to clinical testing and wastewater testing in lab.

**Data analysis**

All samples from the WWTPs and natural water bodies were subjected to concurrent detection via RT-qPCR and the WATER NEWS system. For the samples tested by WATER NEWS, the generated fluorescence signal was compared with the threshold. The threshold was set as the mean of three negative experiments plus three times the standard deviation. If the fluorescence value exceeded this threshold, it was defined as positive; otherwise, it was defined as negative. The threshold for the micro-device was set as the fluorescence value obtained from 10 consecutive negative sample tests plus three times the standard deviation. Positive samples were identified if their signal exceeded this set threshold. Statistical significance was analyzed using a two-tailed *t*-test: ns, *p* > 0.05; *, *p* < 0.05; **, *p* < 0.01; ***, *p* < 0.001; ****, *p* < 0.0001. All experiments were conducted in triplicates, and the mean fluorescence value was used for analysis.

**Supplementary Table 1. crRNAs used in this work.**

| crRNAs | Spacer sequence | Source or reference |
| --- | --- | --- |
| *f3l*-cr1 | UUGGAAAGGUGUUAACCCUG | This study |
| *f3l*-cr2 | ACCGUUAUUAAUGAGUACUG | This study |
| *f3l*-cr3 | ACACCUUUCCAAUAAAUAAU | This study |
| *b6r*-cr1 | ACUGGUUGACACGAUCCGUG | This study |
| *b6r*-cr2 | AACCUCUUCAAUUAGAACAC | This study |
| *b6r*-cr3 | AAGAAAAAUACUCAUUUGGG | This study |
| *miaA*-cr | CCGUCAGGUGUGGGAUUACC | This study |
| *CHS1*-cr | UCGGGUUUGGUACCCACAUC | This study |
| *covid-19*-cr | UCAUCUGGACUGCUAUUGGU | This study |

**Supplementary Table 2.** **PAM and target sequences used in this work.**

| Name | Non-target and target strand sequence |
| --- | --- |
| *f3l*-T1 | NTS: 5’-TTTATTGGAAAGGTGTTAACCCTG-3’  TS: 3’-AAATAACCTTTCCACAATTGGGAC-5’ |
| *f3l*-T2 | NTS: 5’-TGTCACCGTTATTAATGAGTACTG-3’  TS: 3’-ACAGTGGCAATAATTACTCATGAC-5’ |
| *f3l*-T3 | NTS: 5’-GTTAACACCTTTCCAATAAATAAT-3’  TS: 3’- CAATTGTGGAAAGGTTATTTATTA-5’ |
| *b6r*-T1 | NTS: 5’-TTTAACTGGTTGACACGATCCGTG-3’  TS: 3’-AAATTGACCAACTGTGCTAGGCAC-5’ |
| *b6r*-T2 | NTS: 5’- TGTCAACCTCTTCAATTAGAACAC-3’  TS: 3’-ACAGTTGGAGAAGTTAATCTTGTG-5’ |
| *b6r*-T3 | NTS: 5’-GTTAAAGAAAAATACTCATTTGGG-3’  TS: 3’-CAATTTCTTTTTATGAGTAAACCC-5’ |
| *miaA* | NTS: 5’-GTTACCGTCAGGTGTGGGATTACCTGGA-3’  TS: 3’-CAATGGCAGTCCACACCCTAATGGACCT-5’ |
| *CHS1* | NTS: 5’-GTTATCGGGTTTGGTACCCACATC-3’  TS: 3’-CAATAGCCCAAACCATGGGTGTAG-5’ |
| *covid-19-*N | NTS: 5’- TTGGTCATCTGGACTGCTATTGGT-3’  TS: 3’- AACCAGTAGACCTGACGATAACCA-5’ |

**Supplementary Table 3. RPA and RT primers, qPCR primers and probes used in this work.**

| Primers | DNA sequence |
| --- | --- |
| *f3l*-RPA-1F | CCCGCGAAAAATCAATGAGAGAGGATCATAAGTC |
| *f3l*-RPA-1R | TTCAATACGAAAAGACCAATCTCTCCTAGT |
| *b6r*-RPA-1F | GGAATGATACTGTCACGTGTCCTAATGCGG |
| *b6r*-RPA-1R | CCGTACAACTTATATACGAAACACCAATAACC |
| *miaA*-RPA-1F | CCAGAAGTGACTTGCATGCAGGATTACCGT |
| *miaA*-RPA-1R | CCATCCAGTGTAGATGGCTCCAGCTTCGCA |
| *CHS1*- RPA-1F | CCTGTCTTGGACCCCAATGTTATTGTTCTT |
| *CHS1*- RPA-1R | CTTTACCTTTCATCGCTTTAATTTCACCAG |
| *covid-19*-RPA-1F | CCTTAAATTCCCTCGAGGACAAGGCGTTCC |
| *covid-19*-RPA-1R | CCAGTTCCTAGGTAGTAGAAATACCATCTTGG |
| reverse-transcription | GGAAGTCCAGCTTCTGGC |
| *f3l*-qPCR-1F | TTACAGCTCCAACGATACTC |
| *f3l*-qPCR-1R | TCTCTCATTGATTTTTCGCG |
| *b6r* -qPCR-1F | AACATGTGTACGATCTAACG |
| *b6r* -qPCR-1R | AGTTGCTTCTAACGATTCTA |
| *miaA*-qPCR-1F | TGCATGCAGGATTACCGTCT |
| *miaA*-qPCR-1R | CTCCAGCTTCGCAGCCAGGT |
| *CHS1*-qPCR-1F | CCTGTCTTGGACCCCAATGT |
| *CHS1*-qPCR-1R | AATTTCACCAGCAGCCCCTG |
| *covid-19*-qPCR-1F | GGGGAACTTCTCCTGCTAGAAT |
| *covid-19*-qPCR-1R | CAGACATTTTGCTCTCAAGCTG |
| Probe-1 | FAM-TTATT-BHQ1 |
| Probe-2 | FAM-CCCCCCCC-BHQ1 |
| Probe-strip | FAM-CCCCCCCC-Biotin |
| Probe-5A | FAM-AAAAA-BHQ1 |
| Probe-5G | FAM-GGGGG-BHQ1 |
| Probe-5C | FAM-CCCCC-BHQ1 |
| Probe-5T | FAM-TTTTT-BHQ1 |
| qPCR-*f3l*-probe | FAM-TCCTCGTTGGTCTACGACAATGGATGCTGA-BHQ1 |
| qPCR-*b6r*-probe | FAM-AGAATTTGATCCAGTGGATGATGGTCCCGAC-BHQ1 |
| qPCR-*miaA*-probe | TTACCGTCAGGTGTGGGATTACCTGGA |
| qPCR-*CHS1*-probe | CCCGATAACCATGCCATTTATAATCTATGG |
| *covid-19*-probe | FAM- TTGCTGCTGCTTGACAGATT-BHQ1 |

**Supplementary Table 4.** **The Minimum Information for Publication of Quantitative Real-Time PCR Experiments (MIQE) checklist [5].**

| **ITEM TO CHECK** | **IMPORTANCE** | **CHECKLIST** | **WHERE; COMMENT** |
| --- | --- | --- | --- |
| **EXPERIMENTAL DESIGN** |  |  |  |
| Definition of experimental and control groups | **E** | √ | See Methods |
| Number within each group | **E** | √ | 3 |
| Assay carried out by core lab or  investigator's lab? | **D** | √ | Investigator's lab |
| Acknowledgement of authors' contributions | **D** |  |  |
| **SAMPLE** |  |  |  |
| Description | **E** | √ | See Methods |
| Volume/mass of sample processed | **D** |  |  |
| Microdissection or macrodissection | **E** |  | NA |
| Processing procedure | **E** | √ | See Methods |
| If frozen - how and how quickly? | **E** | √ | Use -80 ℃ refrigerator for frozen storage |
| If fixed - with what, how quickly? | **E** |  | NA |
| Sample storage conditions and duration  (esp. for FFPE samples) | **E** | √ | Stored at -80°C for 1 month |
| **NUCLEIC ACID EXTRACTION** |  |  |  |
| Procedure and/or instrumentation | **E** | √ | See Methods |
| Name of kit and details of any modifications | **E** | √ | Soil DNA extraction kit (MPbio Co., China) and RNA extraction kit (Karroten Biotech Co., China) |
| Source of additional reagents used | **D** |  | 50 μg/ml DNase I (Sangon Biotech Co., China) |
| Details of DNase or RNAse treatment | **E** | √ | As it is a quantitative experiment on DNA, enzymes that degrade DNA were not utilized |
| Contamination assessment (DNA or RNA) | **E** | √ | A260/A280 ratios between 1.75 and 2.03 were obtained for all samples of isolated DNA; A260/A280 ratios between 2.06 and 2.21 were obtained for all samples of isolated RNA |
| Nucleic acid quantification | **E** |  |  |
| Instrument and method | **E** | √ | Absorbance at 260 nm using Nanodrop  (Thermo Fisher Scientific, USA) |
| Purity (A260/A280) | **D** | √ | DNA:1.75-2.03  RNA:2.06-2.21 |
| Yield | **D** |  |  |
| RNA integrity method/instrument | **E** | √ | RNA samples were run on a formaldehyde-agarose gel (1.5%) |
| RIN/RQI or Ct of 3' and 5' transcripts | **E** |  |  |
| Electrophoresis traces | **D** |  |  |
| Inhibition testing (Ct dilutions, spike or other) | **E** |  | NA |
| **REVERSE TRANSCRIPTION** |  |  |  |
| Complete reaction conditions | **E** | √ | See Methods |
| Amount of RNA and reaction volume | **E** | √ | 20 µL total volume |
| Priming oligonucleotide (if using GSP)  and concentration | **E** | √ | NA |
| Reverse transcriptase and concentration | **E** | √ | Reverse Transcriptase (5 U/µL) |
| Temperature and time | **E** | √ | 42 ℃, 15 min |
| Manufacturer of reagents and catalogue numbers | **D** | √ | StepOne real-time PCR system (ABI Company, USA) |
| Cts with and without RT | **D** | √ | All Cqs without RT were observed to be above 40 |
| Storage conditions of cDNA | **D** | √ | One-step method does not preserve cDNA |
| **qPCR TARGET INFORMATION** |  |  |  |
| If multiplex, efficiency and LOD of each assay | **E** |  | NA |
| Sequence accession number | **E** | √ | See Methods |
| Location of amplicon | **D** |  |  |
| Amplicon length | **E** | √ | *f3l*: 120 bp  *b6r*: 127 bp  *N*: 99 bp |
| In silico specificity screen (BLAST, etc) | **E** | √ | Done by NCBI BLAST |
| Pseudogenes, retropseudogenes or other homologs? | **D** |  |  |
| Sequence alignment | **D** |  |  |
| Secondary structure analysis of amplicon | **D** |  |  |
| Location of each primer by exon or intron (if applicable) | **E** | √ | NA |
| What splice variants are targeted? | **E** |  | NA |
| **qPCR OLIGONUCLEOTIDES** |  |  |  |
| Primer sequences | **E** | √ | See table S3 |
| RT Primer DB Identification Number | **D** |  |  |
| Probe sequences | **D** | √ | See table S3 |
| Location and identity of any modifications | **E** | √ | See table S3 |
| Manufacturer of oligonucleotides | **D** | √ | Sangon Biotech Co., China |
| Purification method | **D** | √ | PAGE |
| **qPCR PROTOCOL** |  |  |  |
| Complete reaction conditions | **E** | √ | See Methods |
| Reaction volume and amount of cDNA/DNA | **E** | √ | 20 µL total volume |
| Primer, (probe), Mg++ and dNTP | **E** | √ | 1 μL of each primer pair at 10 μM, and 0.2 μL 10 μM TaqMan probe. Mg++ and dNTP were incorporated in Probe Master |
| Polymerase identity and concentration | **E** | √ | Polymerase was incorporated in Probe Master |
| Buffer/kit identity and manufacturer | **E** | √ | AceQ qPCR probe master mix (Nanjing Vazyme Biotech Co., China) |
| Exact chemical constitution of the buffe | **D** |  |  |
| Additives (SYBR Green I, DMSO, etc.) | **E** | √ | Use probe |
| Manufacturer of plates/tubes and catalog  number | **D** | √ | Sangon Biotech Co., China |
| Complete thermocycling parameters | **E** | √ | See Methods |
| Reaction setup (manual/robotic) | **D** | √ | Manual |
| Manufacturer of qPCR instrument | **E** | √ | StepOne real-time PCR system (ABI Company, USA) |
| **qPCR VALIDATION** |  |  |  |
| Evidence of optimisation | **D** |  |  |
| Specificity (gel, sequence, melt, or digest) | **E** | √ | Taqman probe |
| For probe, Ct of the NTC | **E** | √ | Undetectable in all cases |
| Linear dynamic range | **E** | √ | See figure S3 and figure 5 |
| Ct variation at lower limit | **E** | √ | See figure S3 and figure 5 |
| Evidence for limit of detection | **E** | √ | Standard Curve |
| If multiplex, efficiency and LOD of each assay | **E** |  | NA |
| **DATA ANALYSIS** |  |  |  |
| qPCR analysis program (source, version) | **E** | √ | Light Cycler 48 |
| Ct method determination | **E** | √ | Instrument automatic analysis |
| Results of NTCs | **E** | √ | No Ct values were detected in NTCs |
| Justification of number and choice of reference genes | **E** | √ | No reference gene  when using absolute quantification method |
| Description of normalisation method | **E** | √ | Use absolute quantification method |
| Number and concordance of biological replicates | **D** |  |  |
| Number and stage (RT or qPCR) of technical replicates | **E** | √ | n=3 replicates for qPCR and RT-qPCR |
| Statistical methods for result significance | **E** | *√* | *P-value* |
| Ct or raw data submission using RDML | **D** |  |  |
| All required details (E) must accompany the submission of the manuscript. If accessible, favorable data (D) should be included. | | | |

**Supplementary Table 5.** **Sampling locations in Chaohu and Yexi Lakes, Hefei, China.**

| Sampling point | Latitude (N) | Longitude (E) |
| --- | --- | --- |
| RM-1 | 31.693 | 117.405 |
| RM-2 | 31.554 | 117.369 |
| RM-3 | 31.537 | 117.403 |
| RM-4 | 31.612 | 117.792 |
| Yexi lake-1 | 31.841 | 117.251 |
| Yexi lake-2 | 31.841 | 117.250 |

**Supplementary Table 6.** **Sampling sities for SARS-CoV-2 detection.**

| Sampling WWTPs | City | Latitude (N) | Longitude (E) |
| --- | --- | --- | --- |
| WWTP-1 | Chengdu | 30.75° | 103.91° |
| WWTP-2 | Chengdu | 30.57° | 104.27° |
| WWTP-3 | Chengdu | 30.49° | 103.99° |
| WWTP-4 | Xian | 34.39° | 108.85° |
| WWTP-5 | Xian | 34.60° | 109.22° |
| WWTP-6 | Qingdao | 36.28° | 120.10° |
| WWTP-7 | Qingdao | 36.25° | 120.03° |
| WWTP-8 | Qingdao | 36.23° | 120.18° |
| WWTP-9 | Jinzhou | 41.11° | 121.20° |
| WWTP-10 | Jinzhou | 41.09° | 121.13° |
| WWTP-11 | Luoyang | 34.65° | 112.43° |
| WWTP-12 | Luoyang | 34.70° | 112.54° |
| WWTP-13 | Guangzhou | 23.17° | 113.79° |
| WWTP-14 | Guangzhou | 23.36° | 113.19° |
| WWTP-15 | Guangzhou | 23.13° | 113.65° |
| WWTP-16 | Beijing | 40.09° | 116.22° |
| WWTP-17 | Beijing | 40.15° | 116.30° |
| WWTP-18 | Beijing | 39.78° | 116.19° |
| WWTP-19 | Sanhe | 39.95° | 116.81° |
| WWTP-20 | Sanhe | 39.99° | 116.21° |
| WWTP-21 | Sanhe | 39.96° | 116.82° |
| WWTP-22 | Kunming | 24.97° | 102.45° |
| WWTP-23 | Kunming | 25.04° | 102.88° |
| WWTP-24 | Kunming | 24.95° | 102.58° |
| WWTP-25 | Wuxi | 31.53° | 120.37° |

**Supplementary Table 7.** **Daily treatment scale and process of WWTPs.**

| **WWTPs** | **Treatment scale (m^3^/d)** | **Core technology** |
| --- | --- | --- |
| 1 | 100,000 | AAO |
| 2 | 100,000 | multi-stage AO |
| 3 | 100,000 | AAO |
| 4 | 50,000 | AAO |
| 5 | 50,000 | AAO |
| 6 | 150,000 | AAO |
| 7 | 150,000 | AAO |
| 8 | 90,000 | AAO |
| 9 | 200,000 | AAO |
| 10 | 50,000 | AAO |
| 11 | 300,000 | AAO |
| 12 | 250,000 | AAO |
| 13 | 150,000 | AAO |
| 14 | 200,000 | AAO |
| 15 | 50,000 | multi-stage AO |
| 16 | 80,000 | multi-stage AO |
| 17 | 60,000 | AAO |
| 18 | 50,000 | AAO |
| 19 | 50,000 | AAO,MBR |
| 20 | 50,000 | MBR,A2O |
| 21 | 50,000 | AAO |
| 22 | 50,000 | SBR |
| 23 | 40,000 | AAO |
| 24 | 12,500 | AAO |
| 25 | 150,000 | AAO |

AAO: Anaerobic/Anoxic/Oxic; AO: Anoxic/Oxic;

MBR: Membrane bioreactor; SBR: Sequential batch reactor.

**Supplementary Table 8.** **Procurement prices and numbers for equipment in 2024 US$, allocation to SARS-CoV-2 testing, and assumed useful life.**

| Capital equipment： | Full procurement price (US$) | Numbers | Allocation to  SARS-CoV-2 testing | | Assumed useful life (years) | |
| --- | --- | --- | --- | --- | --- | --- |
| **Nucleic acid testing laboratory for human samples** | | | | | |  |
| Quantitative PCR (RT-qPCR) | 48,344.5 | 6 | | 50% | | 10 |
| PCR mini spinner | 386.8 | 1 | | 50% | | 10 |
| Vortex | 828.8 | 1 | | 50% | | 10 |
| Microcentrifuge for RNA extraction | 1,657.5 | 1 | | 50% | | 10 |
| Pipet-aid (different ranges ) | 78.5 | 6 | | 50% | | 2 |
| Freeze –20 ℃  (Refrigerator) | 635.1 | 1 | | 50% | | 10 |
| Freeze –80 ℃  (Refrigerator) | 6,388.8 | 1 | | 50% | | 10 |
| Venue rent/month | 4974.0 | 1 | | 100% | | 12 |
| **Monthly cost** | **1,674.2** | | | | | |
| **Nucleic acid testing laboratory for wastewater samples** | | | | | |  |
| Quantitative PCR (RT-qPCR) | 48,344.5 | 1 | | 100% | | 10 |
| PCR mini spinner | 386.8 | 1 | | 100% | | 10 |
| Vortex | 828.8 | 1 | | 100% | | 10 |
| Microcentrifuge for RNA extraction | 1,657.5 | 1 | | 100% | | 10 |
| Large centrifuge | 10,934.1 | 1 | | 100% | | 10 |
| Electronic weighing balance | 592.3 | 1 | | 100% | | 10 |
| Autoclave | 4,212.3 | 1 | | 100% | | 10 |
| Pipet-aid | 78.5 | 3 | | 100% | | 2 |
| Freeze –20 ℃  (Refrigerator) | 635.1 | 1 | | 100% | | 10 |
| Freeze –80 ℃  (Refrigerator) | 6,388.8 | 1 | | 100% | | 10 |
| Beaker 250mL (sampling cup) | 1.3 | 25 | | 100% | | 1 |
| Sampler for wastewater | 52.6 | 25 | | 100% | | 1 |
| Venue rent/month | 4974.0 | 1 | | 100% | | 12 |
| **Monthly cost** | **1,153.1** | | | | | |
| **Field wastewater surveillance** | | | | | |  |
| Field diagnostic device (micro-device) | 110 | 25 | | 100% | | 2 |
| Beaker 250mL (sampling cup) | 1.3 | 25 | | 100% | | 1 |
| Pipet-aid |  |  | |  | |  |
| Sampler for wastewater | 52.6 | 25 | | 100% | | 1 |
| **Monthly cost** | **170.7** | | | | | |

**Note:** To determine the minimum equipment cost required for laboratory-based human sample testing for SARS-CoV-2, calculations were based on the need for 100 to 1,000 tests per million people [4, 6]. Using Hong Kong as a reference, with approximately 25 wastewater monitoring points covering 1 million people, we calculated the associated costs [7]. Monthly cost = (Full procurement price × Numbers × Numbers Allocation to SARS-CoV-2)/ Assumed useful life.

**Supplementary Table 9.** **Cost analysis of consumables and reagents.**

| Consumables and reagents | **Human samples in lab (US$)** | **Wastewater samples in lab (US$)** | **Field wastewater surveillance (US$)** |
| --- | --- | --- | --- |
| Nucleic acid collection tube | 0.5 | NA | NA |
| Wastewater collection bottle 500 mL | NA | 2.5 | 2.5 |
| PEG mixture for concentration | NA | 0.4 | 0.4 |
| Viral RNA Mini Extraction Kit | 2.3 | 2.3 | NA |
| One-step detection reagent (RT-qPCR) | 2.2 | 2.2 | NA |
| One-pot detection reagent (RT-RPA-CRISPR)[8] | NA | NA | 2.5 |
| PCR plate and  Pipette tip | 0.5 | 0.5 | 0.3 |
| Rnase/Dnase free water and tubes | 0.1 | 0.2 | 0.2 |
| Filter and membrane | NA | NA | 0.4 |
| Mag-MK Virus RNA Extraction Kit | NA | NA | 2.1 |
| Other reagents and consumables | 0.3 | 0.2 | 0.1 |
| **Single-sample cost** | **5.9** | **8.3** | **8.5** |
| **Estimated number**  **of samples** | **100-1000** | **25** | **25** |
| **Total cost** | **590-5,900** | **207.5** | **212.5** |
| **Monthly cost** | **17,700-177,000** | **3,112.5** | **3,187.5** |

**Note:** Total cost = Single-sample cost × Estimated number of samples. Human sample testing was conducted daily, while wastewater monitoring was performed every other day. Therefore, Monthly cost (human samples) = Total cost × 30. Monthly cost (wastewater samples) = Total cost × 15.

**Supplementary Table 10.** **Monthly salary and benefits for key personnel in 2024 US$ and allocation of monthly salary to SARS-CoV-2 testing.**

| Job | Numbers | Monthly salary and benefits | Allocation of monthly salary to testing |
| --- | --- | --- | --- |
| **Human samples testing** | | | |
| Nucleic acid sampling worker | 10 | 966.4 | 100% |
| Transport driver | 1 | 1,380.6 | 100% |
| Laboratory Scientist | 1 | 2,761.1 | 100% |
| Technical person | 2 | 1,173.5 | 100% |
| **Monthly cost** | **16,152.7** | | |
| **Wastewater samples testing in lab** | | | |
| Wastewater sampling worker | 10 | 1,035.4 | 10% |
| Transport driver | 1 | 1,380.6 | 50% |
| Laboratory Scientist | 1 | 2,761.1 | 50% |
| Technical person | 2 | 1,173.5 | 50% |
| **Monthly cost** | **4,279.8** | | |
| **Field wastewater surveillance** | | | |
| Wastewater sampling and testing worker | 10 | 1,380.6 | 10% |
| **Monthly cost** | **1,380.6** | | |

**Note:** Ten nucleic acid sampling points were need for every 1 million residents. Monthly salaries and the time spent on SARS-CoV-2 sampling and testing were obtained through interviews with staff. Since wastewater samples were collected and tested every other day, the time spent by transport drivers and laboratory technicians was consequently halved. Monthly cost = Numbers × Monthly salary and benefits × Allocation of monthly salary to testing.

**
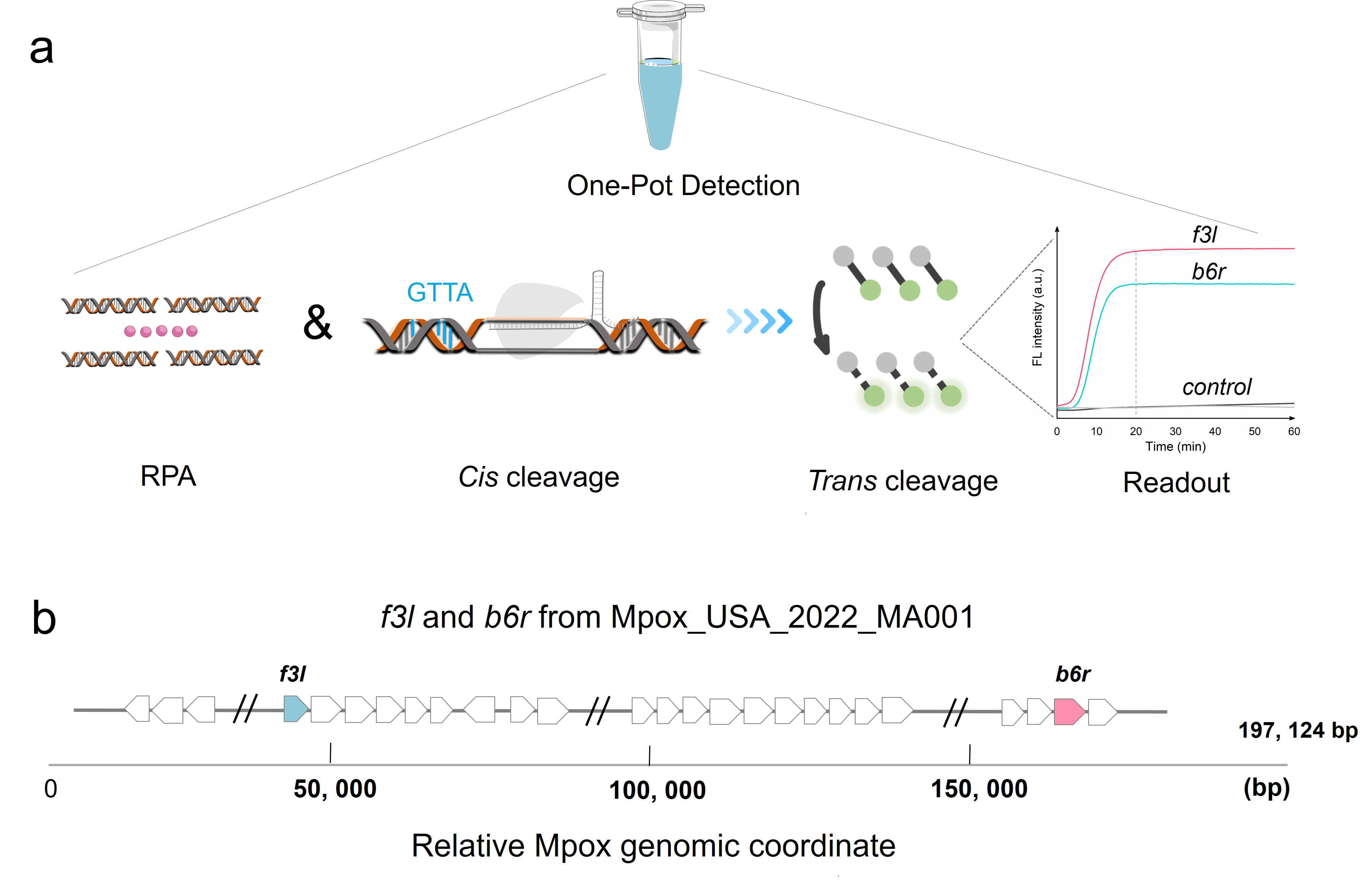
**

**Supplementary Fig. 1****.** **Design of one-pot assay for mpox detection. a**, Workflow for one-pot assay for mpox detection. **b**, Conserved genes (*f3l* and *b6r*) in the mpox genome.

The schematics shown in supplementary fig. 1a were created by figdraw.com.

**
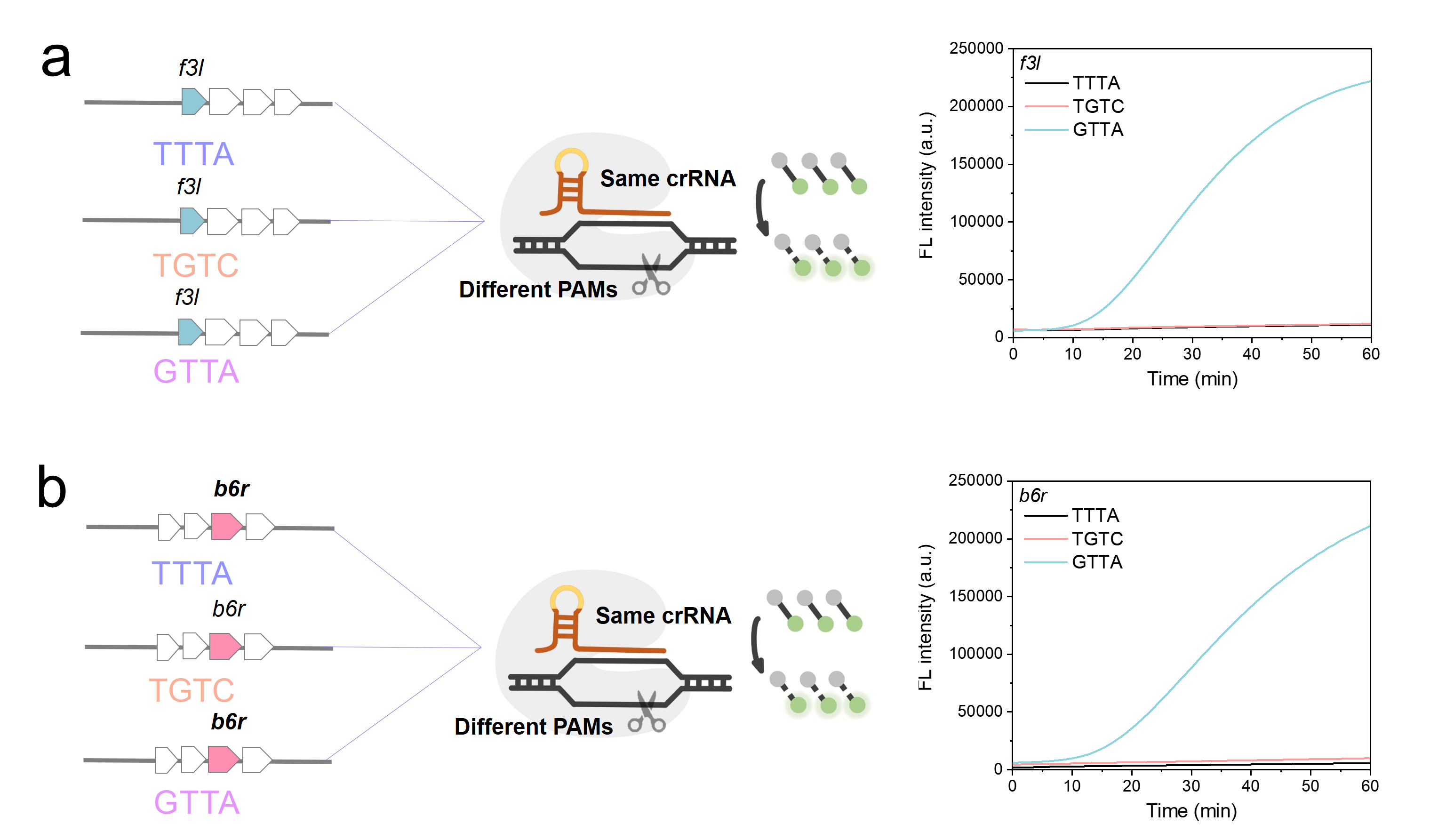
**

**Supplementary Fig. 2.** **Using the same crRNA to target three DNAs with only differences in their PAM for a one-pot assay. a**, Three plasmids containing the *f3l* gene were designed with TTTA, TGTC, and GTTA as their respective PAMs at the same location, and then subjected to a one-pot assay using the same crRNA. **b**, Under identical conditions, the target gene was *b6r*.


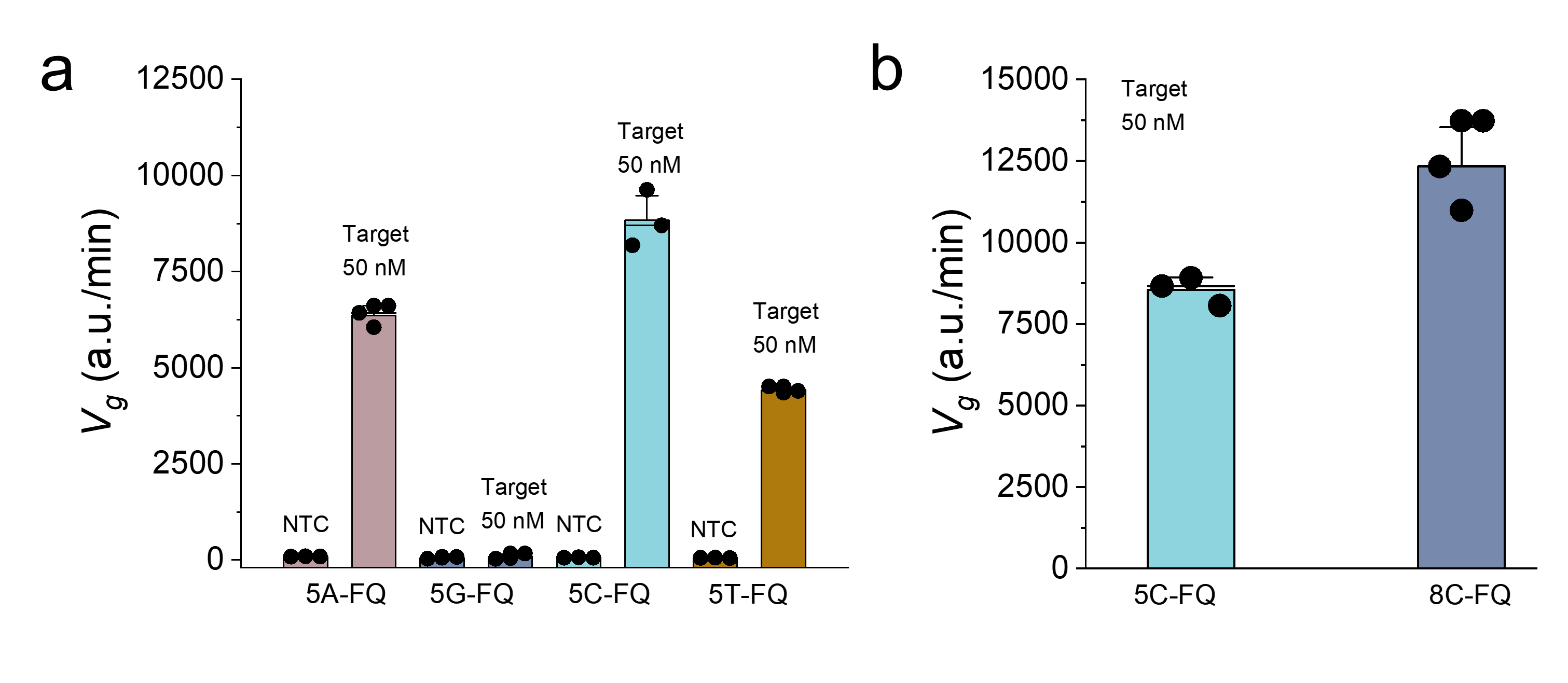


**Supplementary Fig. 3.** **Characterization of *trans*-cleavage activity for fluorescent probes with distinct probe sequences. a-b,** Fluorescent reporting probes containing 5A (AAAAA), 5G (GGGGG), 5C (CCCCC), 5T (TTTTT), and 8C (CCCCCCCC) sequences were evaluated for their *trans*-cleavage activity rates under stimulation by 50 nM *f3l* gene activator.


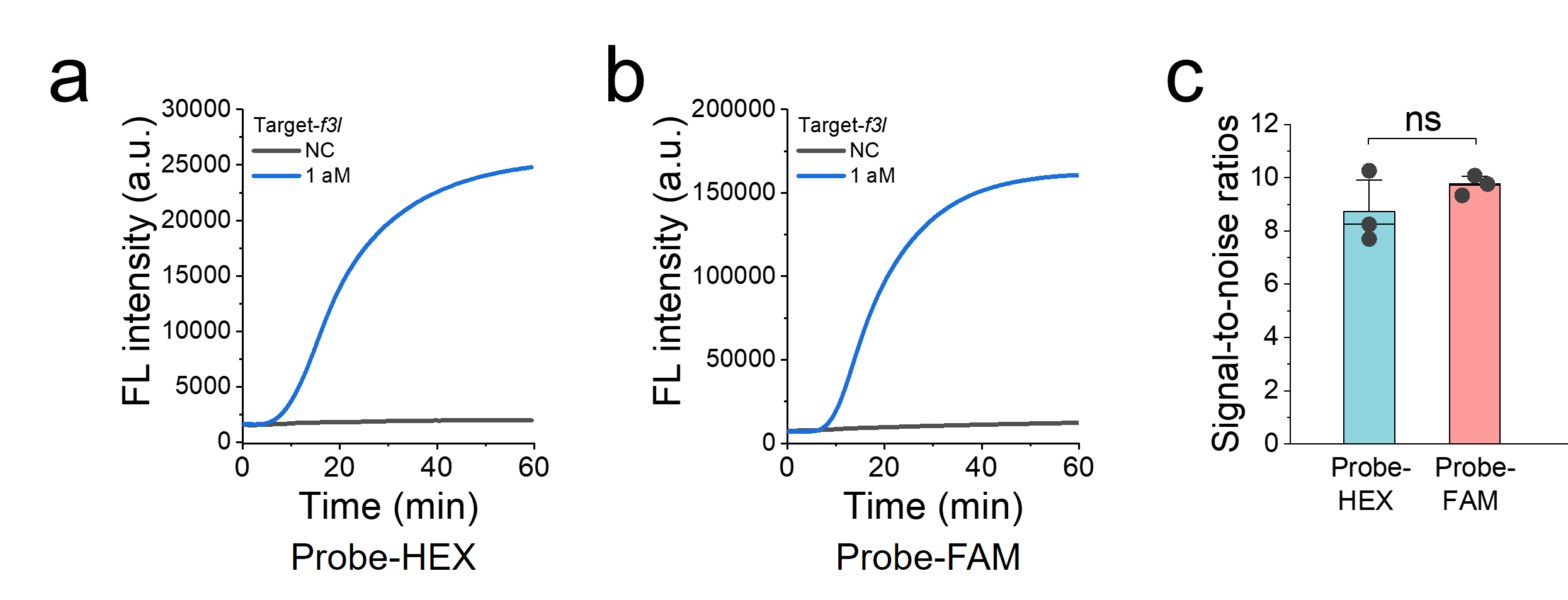


**Supplementary Fig. 4.** **Characterization of fluorophore-labeled probes.** **a-b,** Comparison of one-pot detection performance using two different fluorophores: (a) HEX-labeled probe (HEX-CCCCCCCC-BHQ); **b,** FAM-labeled probe (FAM-CCCCCCCC-BHQ); (c) Signal-to-noise ratio comparison between the two probes.

**
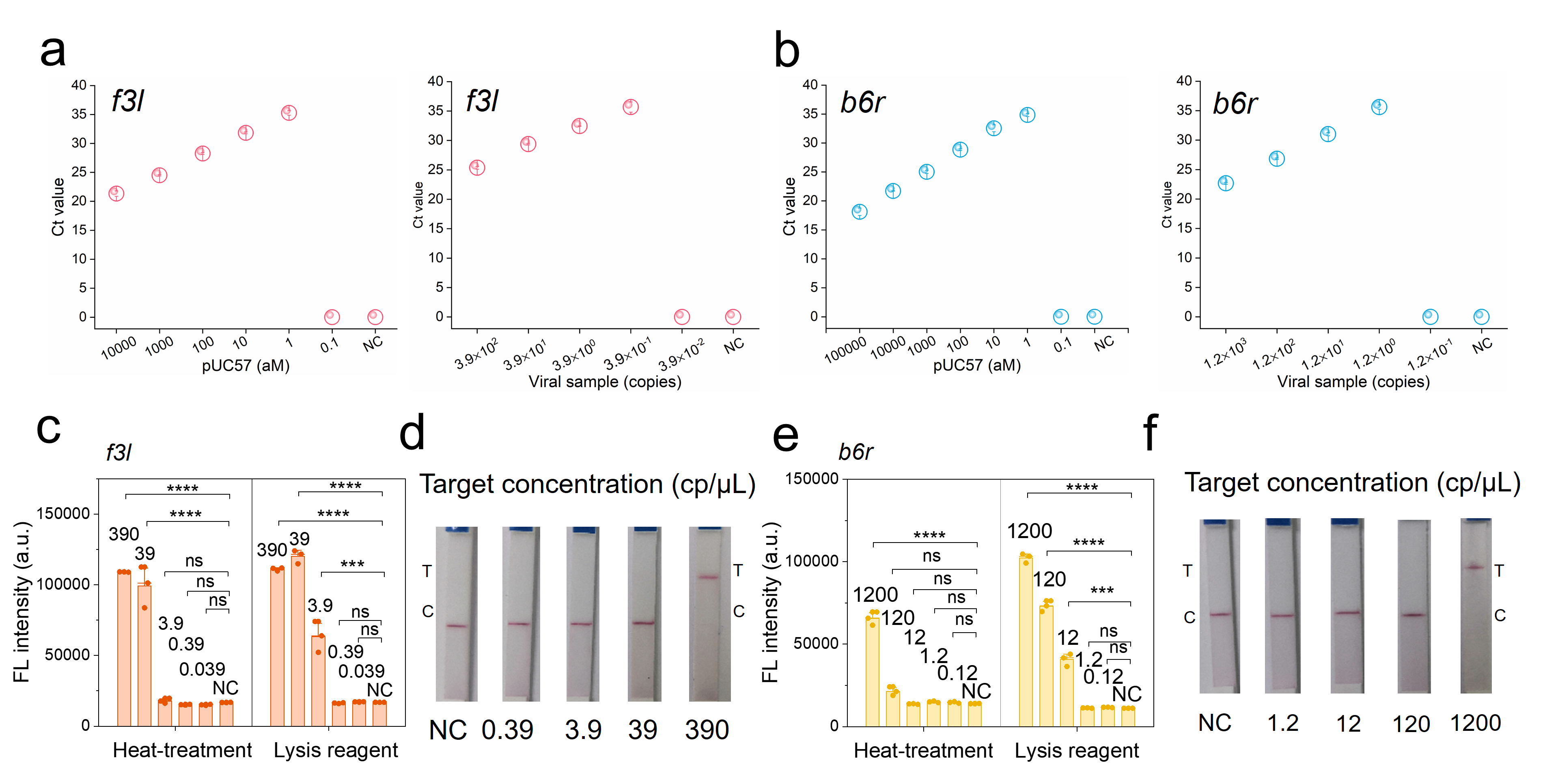
**

**Supplementary Fig. 5.** **Comparison of the LoD with fluorescence and lateral flow strip. a-b**, qPCR standard curves using pUC57-*f3l* or pUC57-*b6r* as plasmid standard. Based on the Ct value and standard curve, the copy number of pseudoviruses with the *f3l* or *b6r* gene was calculated. **c**,**e**, Comparison of the release efficiency and the LoD of *f3l* (**c**) and *b6r* (**e**) from pseudoviruses with the lysis reagents (25 °C, 1 min) and pyrolysis (95 °C, 5 mins) based on fluorescence readout. The fluorescence values at 20 minutes of the one-pot reaction were compared. **d**,**f**, Quantification of the LoD of WATER NEWS with different concentrations of mpox DNA (**d**: *f3l* gene, **f**: *b6r* gene) released from lysis reagent-treated pseudoviruses samples using the lateral-flow based colorimetric readout. Statistical significance was analyzed using a two-tailed t-test: ns, *p* > 0.05; *, *p* < 0.05; **, *p* < 0.01; ***, *p* < 0.001; ****, *p* < 0.0001.


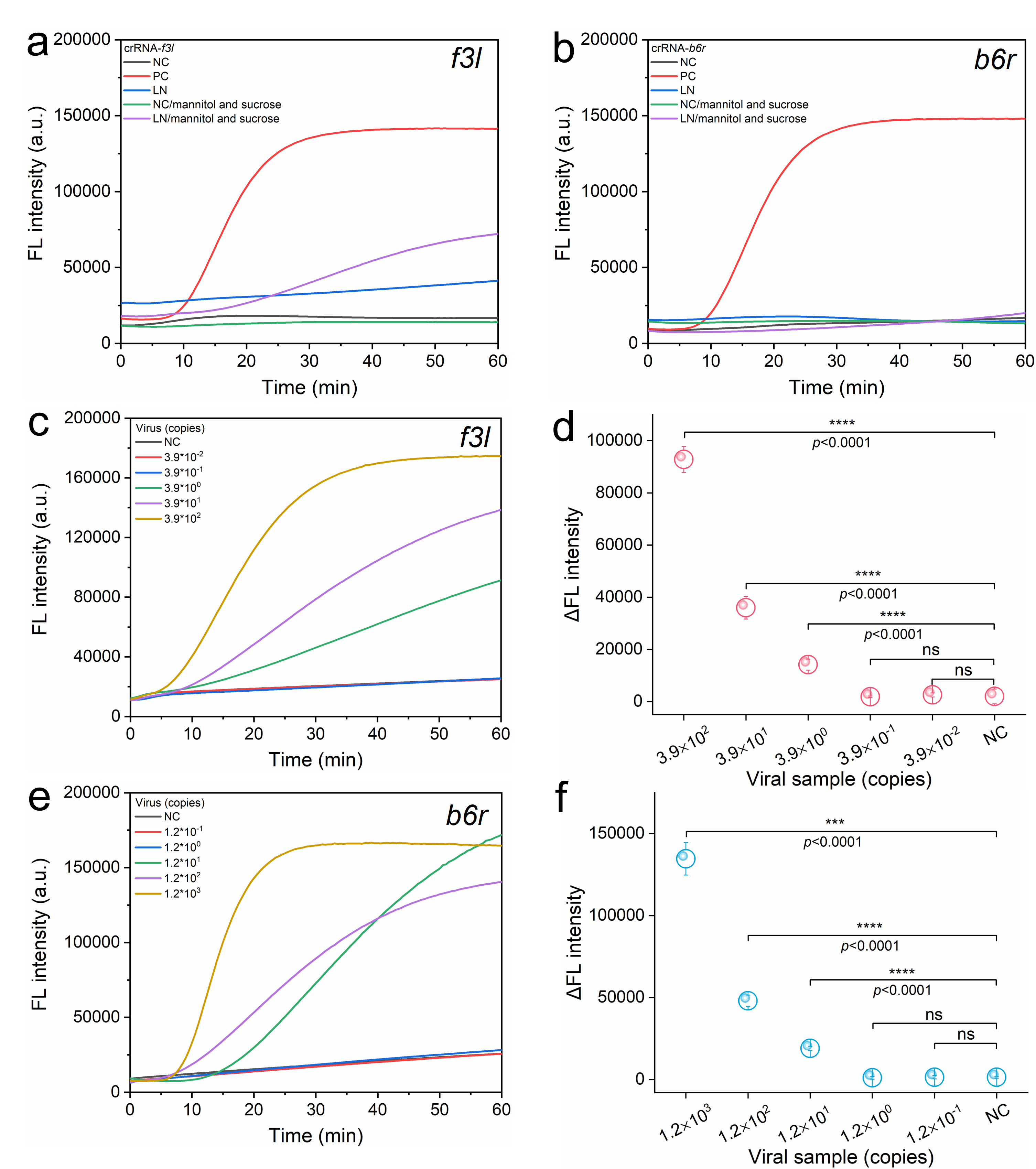


**Supplementary Fig. 6. Fluorescence curves of** **WATER NEWS assays using different lyophilization methods. a-b**, The WATER NEWS assays with lyophilized powder by liquid nitrogen freezing. The roles of mannitol and sucrose on lyophilization were also determined. **c-f**, Characterize the LoD of WATER NEWS assays after freezing at -80 °C for two hours before liquid nitrogen freezing. The fluorescence values at 20 minutes of the one-pot reaction were compared after subtracting the initial fluorescence values. Statistical significance was analyzed using a two-tailed t-test: ns, *p* > 0.05; *, *p* < 0.05; **, *p* < 0.01; ***, *p* < 0.001; ****, *p* < 0.0001.


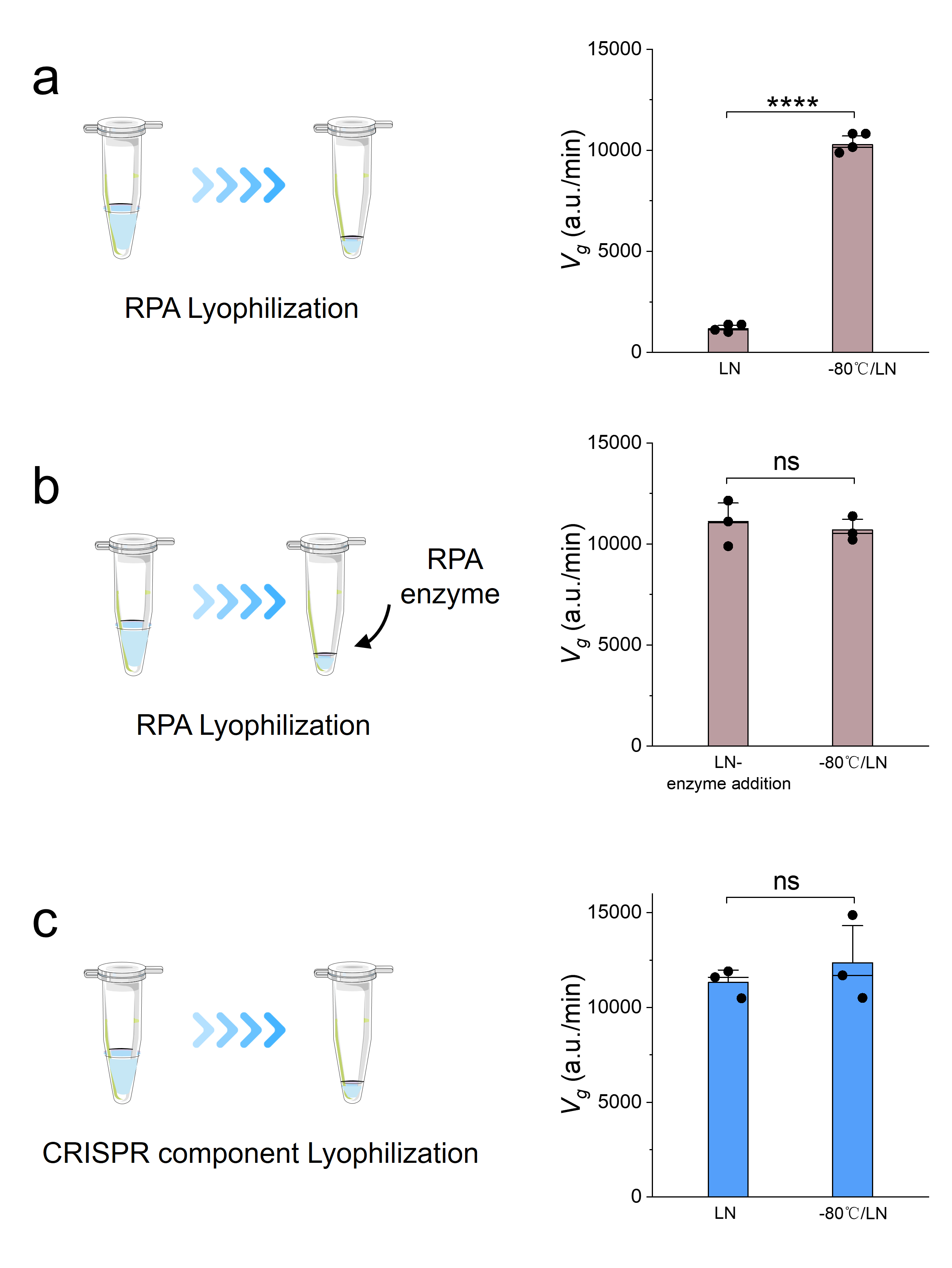


**Supplementary Fig. 7. Lyophilization of key components. a,** RPA reagents were lyophilized either by direct freezing in liquid nitrogen or after pre-cooling at –80 °C for 2 h followed by liquid nitrogen freezing. The resulting lyophilized powders were used for amplification, and the *trans*-cleavage activity of the amplification products on Cas12a was evaluated. **b,** A mixture of enzymes required for RPA amplification was added to the lyophilized powder obtained via direct liquid nitrogen freezing of RPA components, and the *trans*-cleavage activity was assessed. **c.** CRISPR components were lyophilized either by direct freezing in liquid nitrogen or after 2-h pre-cooling at –80 °C followed by liquid nitrogen freezing, and the *trans*-cleavage activity of the resulting powders was directly tested.


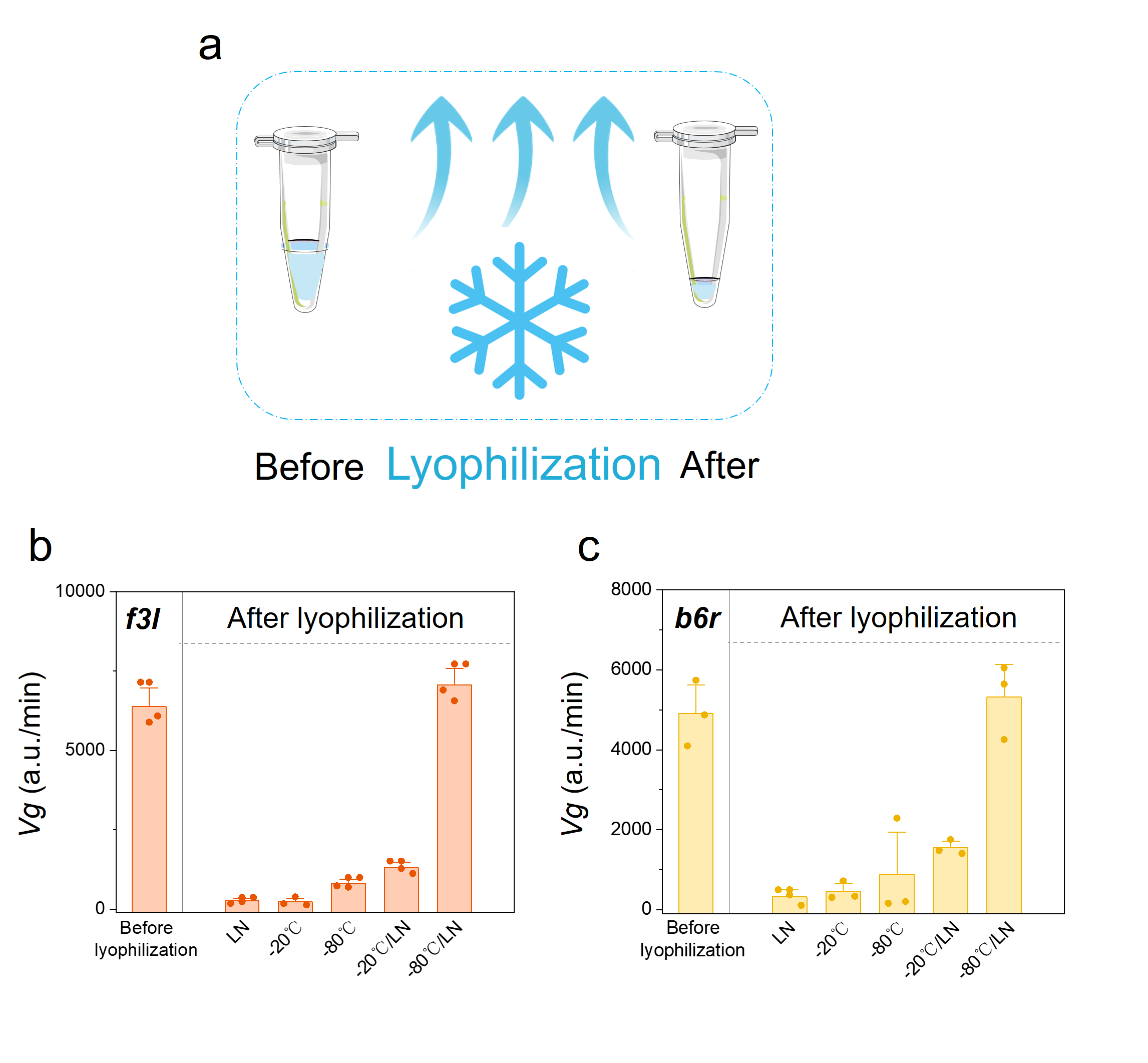


**Supplementary Fig. 8.** **Fluorescence cleavage rate of WATER NEWS assays using different lyophilization methods. a**, Schematic of the lyophilizing one-pot reagents. **b-c**, One-pot fluorescence cleavage rate on pseudoviruses (*f3l*-3.9*10^2^ cp/μl and *b6r*-1.2*10^3^ cp/μl) before and after lyophilization using the different freezing methods. *Vg* (cleavage rate)=FL_20 min_- FL_0 min_/20min. The schematics shown in supplementary fig. 5a were created by figdraw.com.


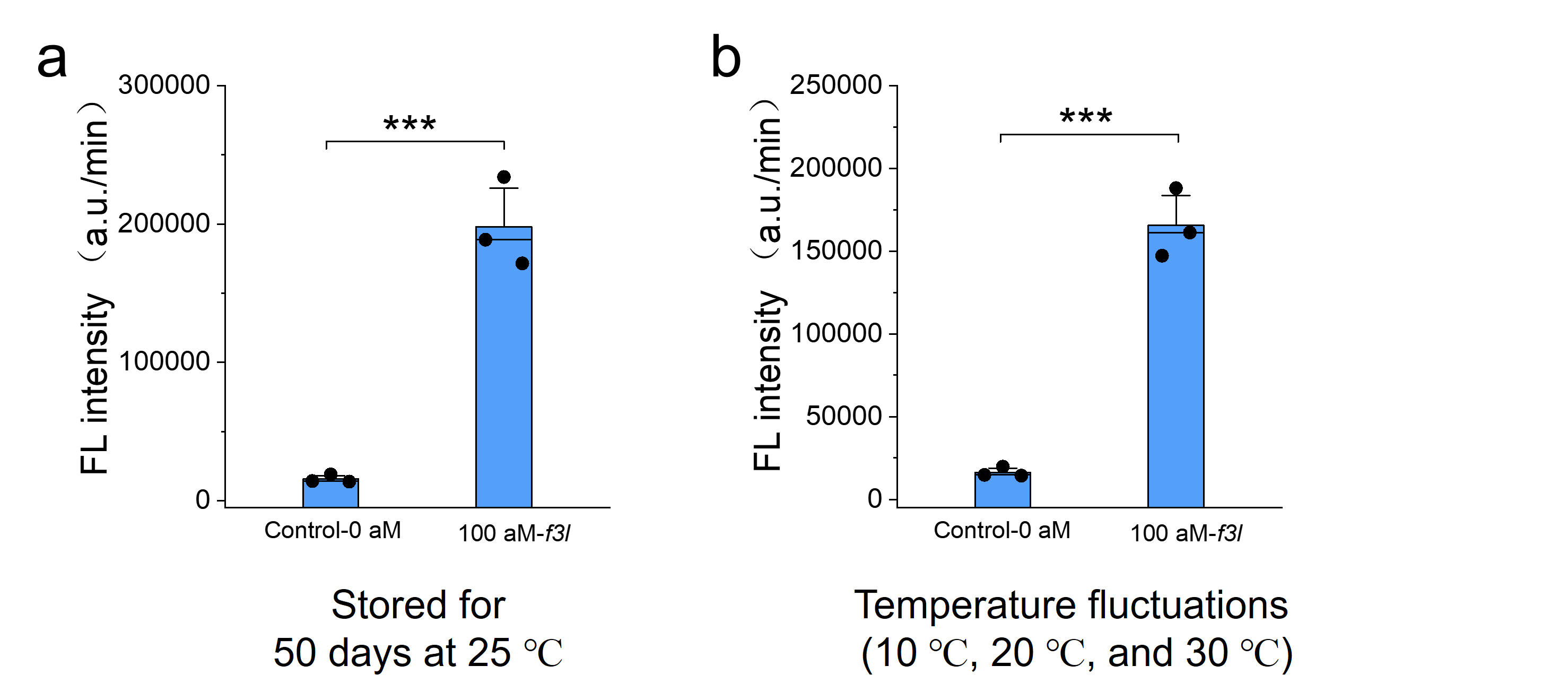


**Supplementary Fig. 9. Stability evaluation of lyophilized reagents. a,** The lyophilized reagents were stored for 50 days, and their performance in one-pot detection assays was assessed to evaluate long-term stability. **b,** To investigate the impact of temperature fluctuations, the lyophilized reagents were stored in the dark at 10 °C, 20 °C, and 30 °C for 1 week each, followed by evaluation of the stability in one-pot detection assays.


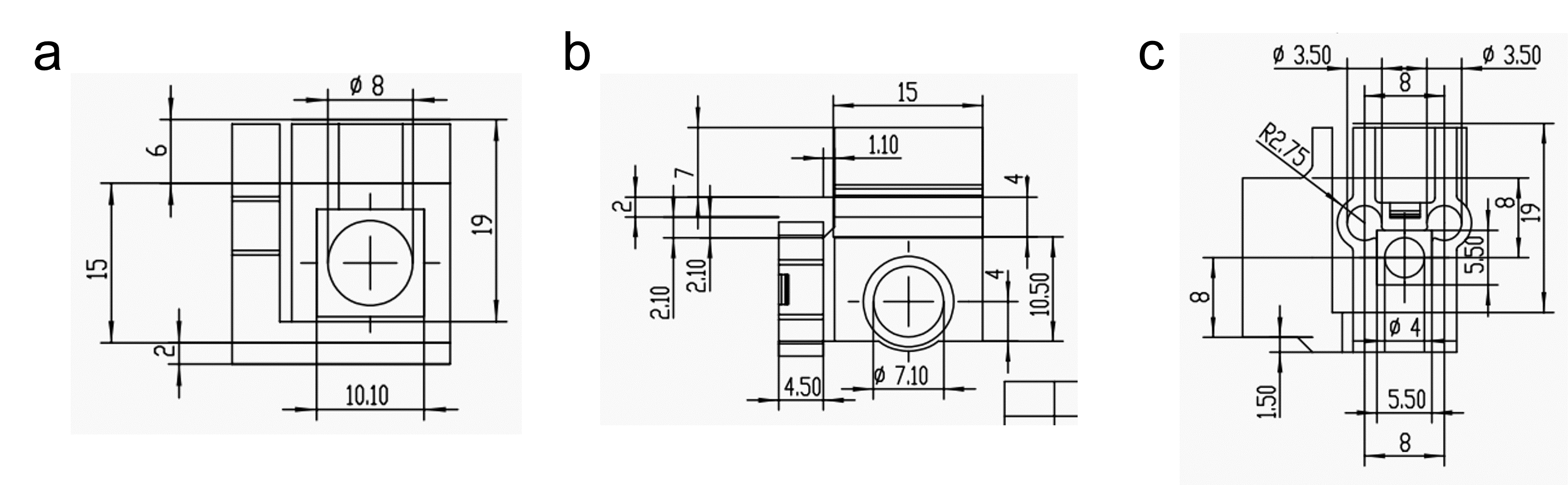


**Supplementary Fig. 10.** **​Orthographic views of the portable device.​​ a-c,** Three orthographic views of the CAD model.


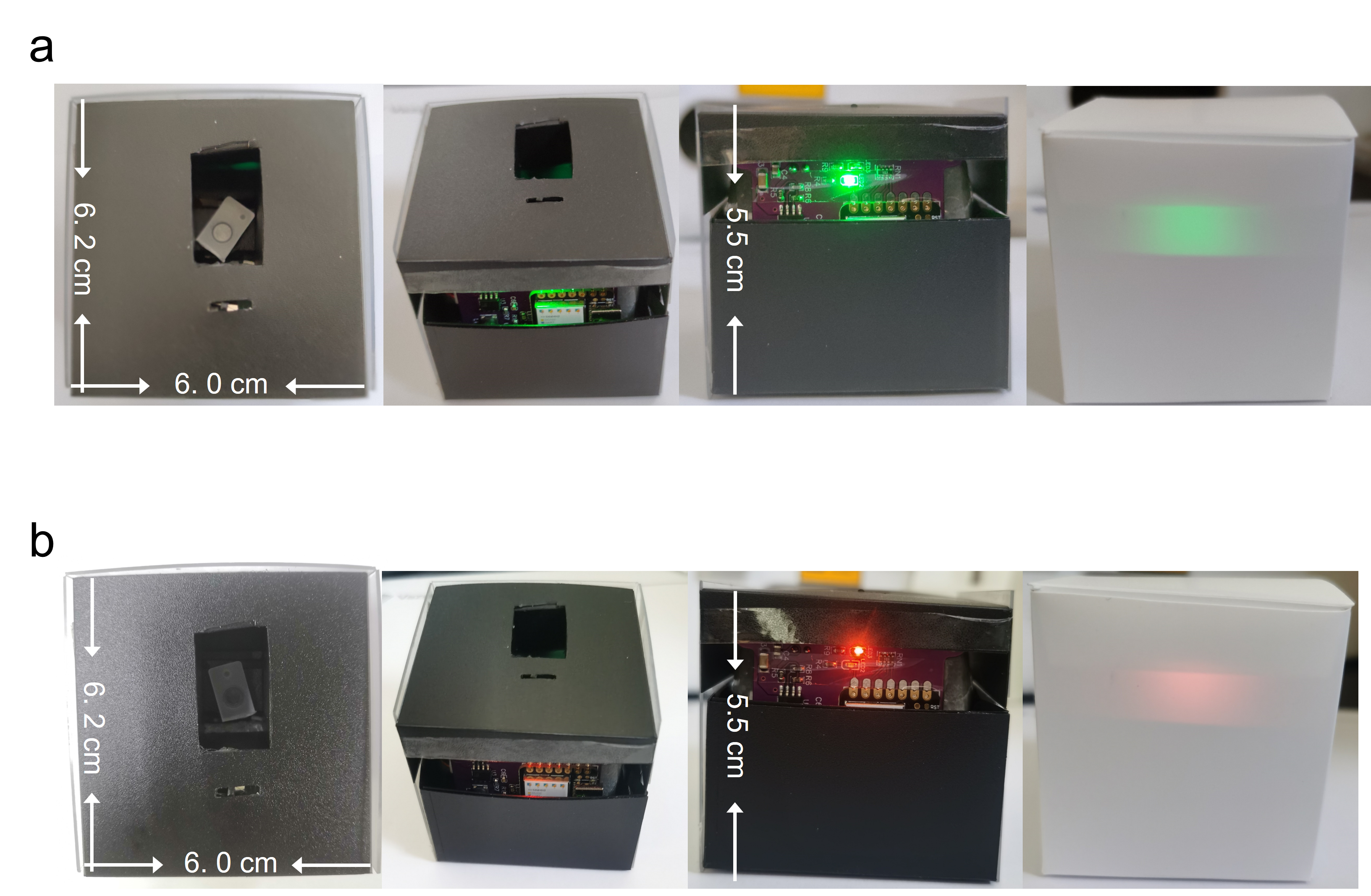


**.Supplementary Fig. 11.** **Schematic diagram of our portable device.** The negative (**a**) and positive samples (**b**) were detected with this Rubik's Cube-size device.


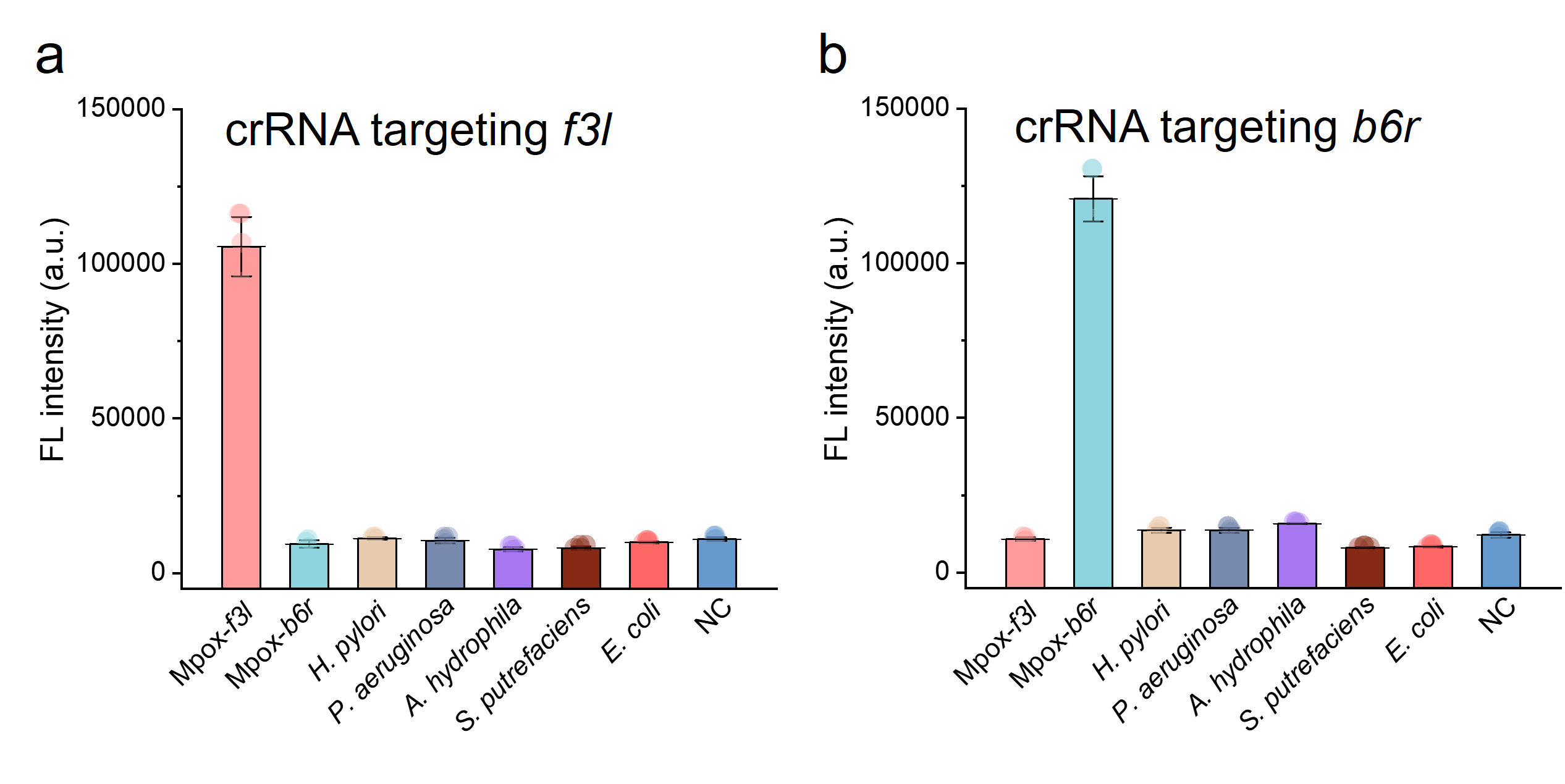


**Supplementary Fig. 12.** **Evaluation of specificity for mpox detection using WATER NEWS.** **a-b**, Using a one-pot detection system targeting the *f3l* (**a**)and *b6r* (**b**) genes, we tested pseudoviruses with the *f3l* or *b6r* gene, *Helicobacter pylori*, *Pseudomonas aeruginosa* PAO1, *Aeromonas hydrophila* ATCC 7966, *Shewanella putrefaciens* CN32, and *Escherichia coli* MG1655. *Helicobacter pylori* was purchased from the Guangdong Microbial Culture Collection Center (GDMCC), with the accession number GDMCC 1.2654.

**
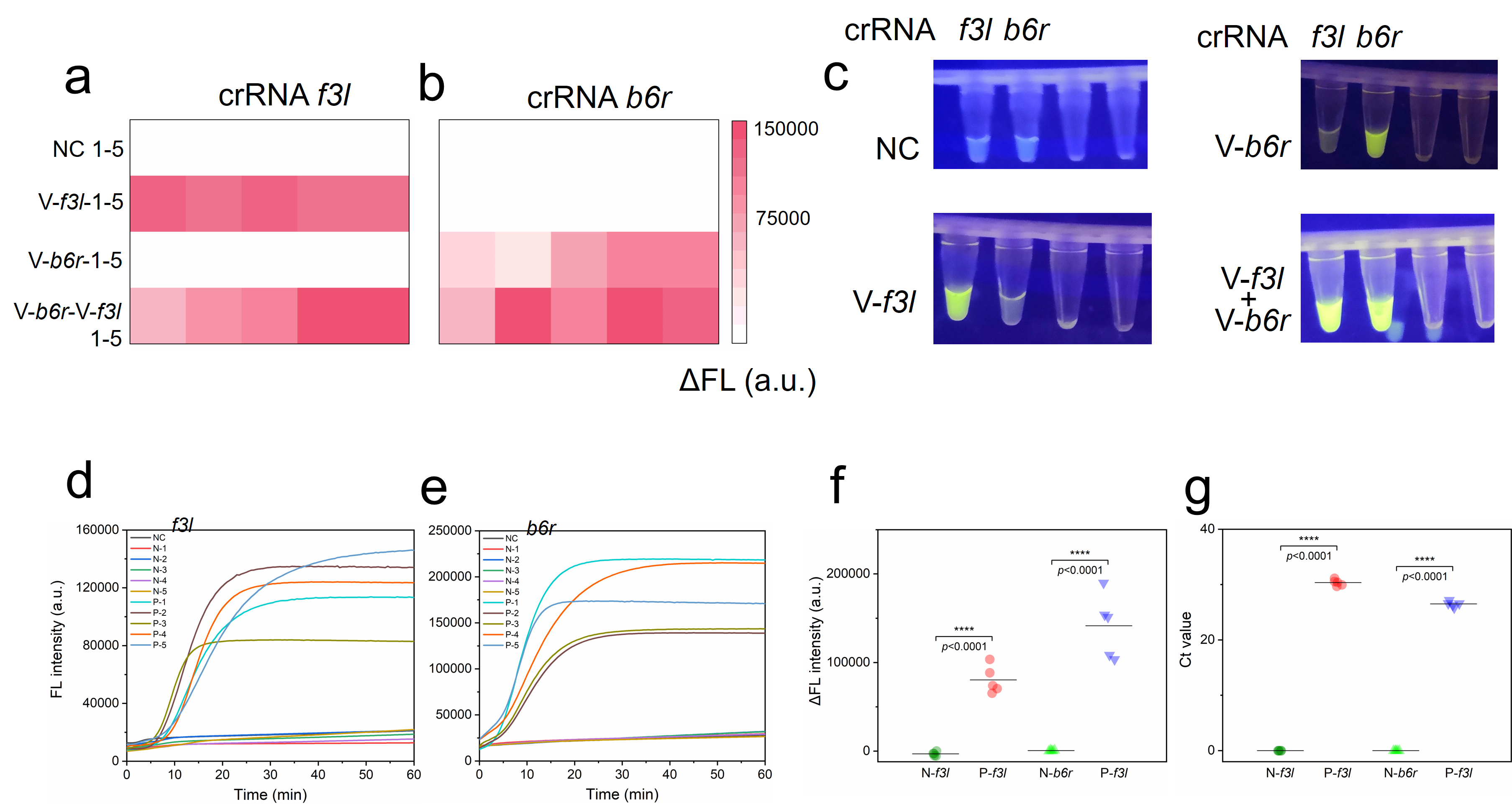
**

**Supplementary Fig. 13.** **Mpox detection in saliva using WATER NEWS.** **a-c**, Crossover experiment. The WATER NEWS assay with crRNA targeting *f3l* could specifically recognize the pseudoviruses containing *f3l* rather than the pseudoviruses containing *b6r*, and vice versa. **d-f**, Mpox detection in saliva samples using WATER NEWS assays. We collected saliva samples from 5 volunteers and divide them into different experimental groups. A portion of the saliva samples was mixed with pseudoviruses containing *f3l* or *b6r* as positive experimental groups, while the rest of saliva samples without pseudoviruses were used as negative experimental groups. The fluorescence values at 20 min of the one-pot reaction were compared. **g**, Negative and positive samples in WATER NEWS assays were confirmed using qPCR. Virus loads: *f3l*-3.9*10^2^ cp/μL and *b6r*-1.2*10^3^ cp/μL. Statistical significance was analyzed using a two-tailed t-test: ns, *p* > 0.05; *, *p* < 0.05; **, *p* < 0.01; ***, *p* < 0.001; ****, *p* < 0.0001.


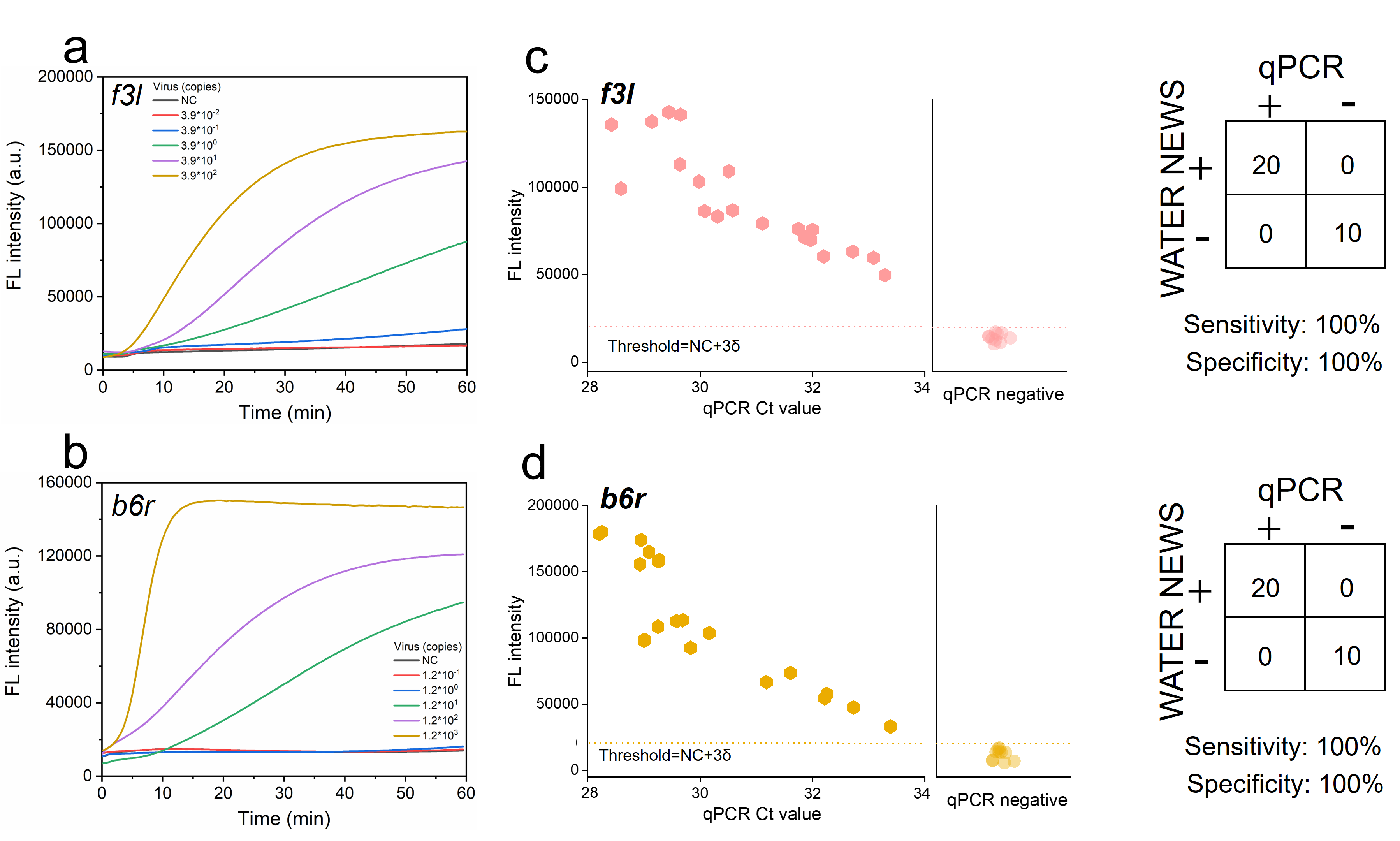


**Supplementary Fig. 14.** **Mpox detection in saliva samples with various Ct values using WATER NEWS assays.** **a-b**, The LoD of the WATER NEWS system when testing saliva samples. **c-d**, We prepared saliva samples with varying Ct values, which were mixed with different concentrations of two pseudoviruses (*f3l* or *b6r*). Among these, 20 samples were designated as positive, and 10 as negative. All samples were subsequently tested using the WATER NEWS. The threshold was set as the mean of three negative experiments plus three times the standard deviation. If the fluorescence value exceeded this threshold, it was defined as positive; otherwise, it was defined as negative.


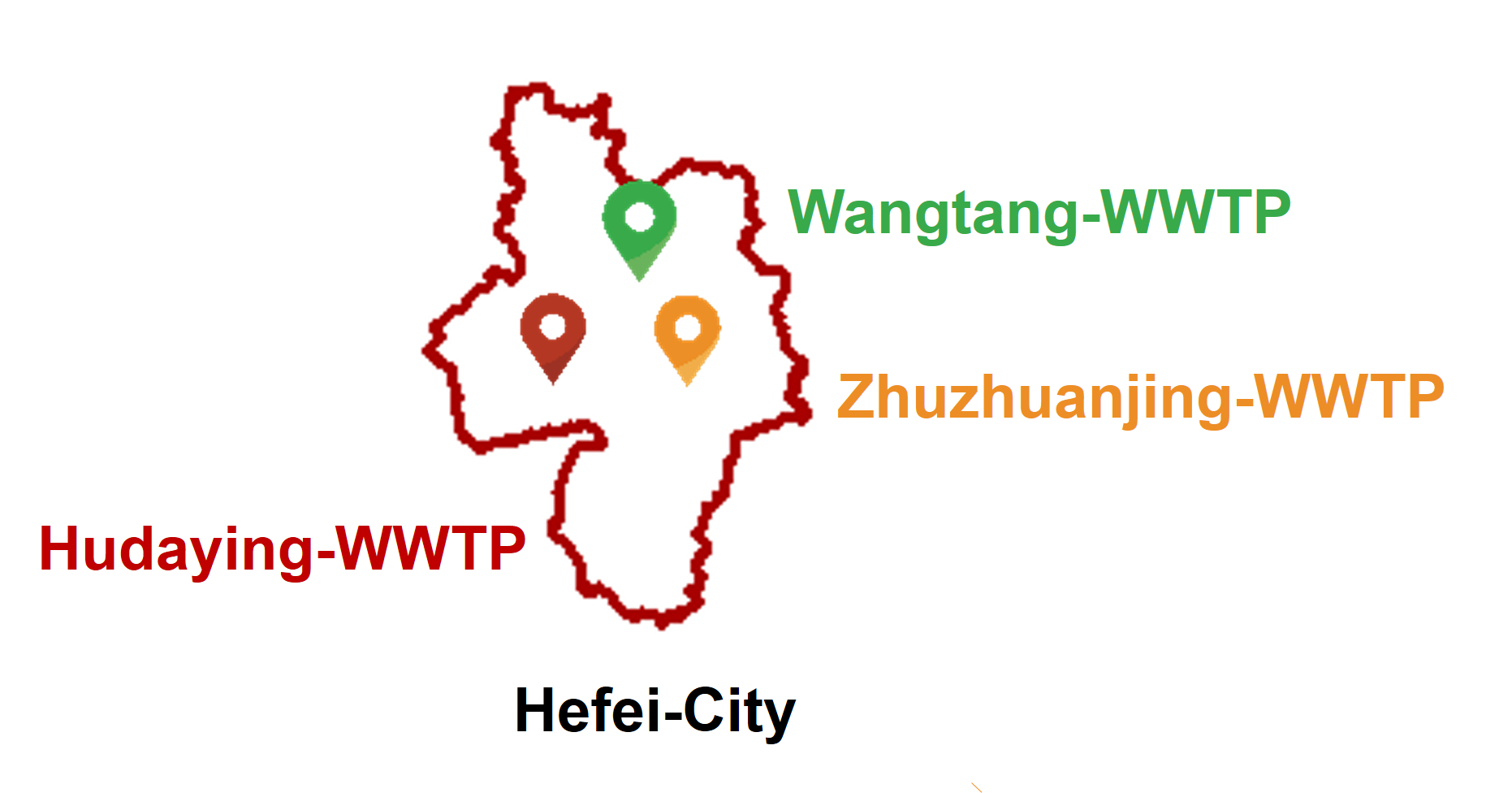


**Supplementary Fig. 15. Map of Hefei city highlighting the three selected WWTPs**. The map illustrates sampling and testing conducted at three WWTPs located in different districts of Hefei City.


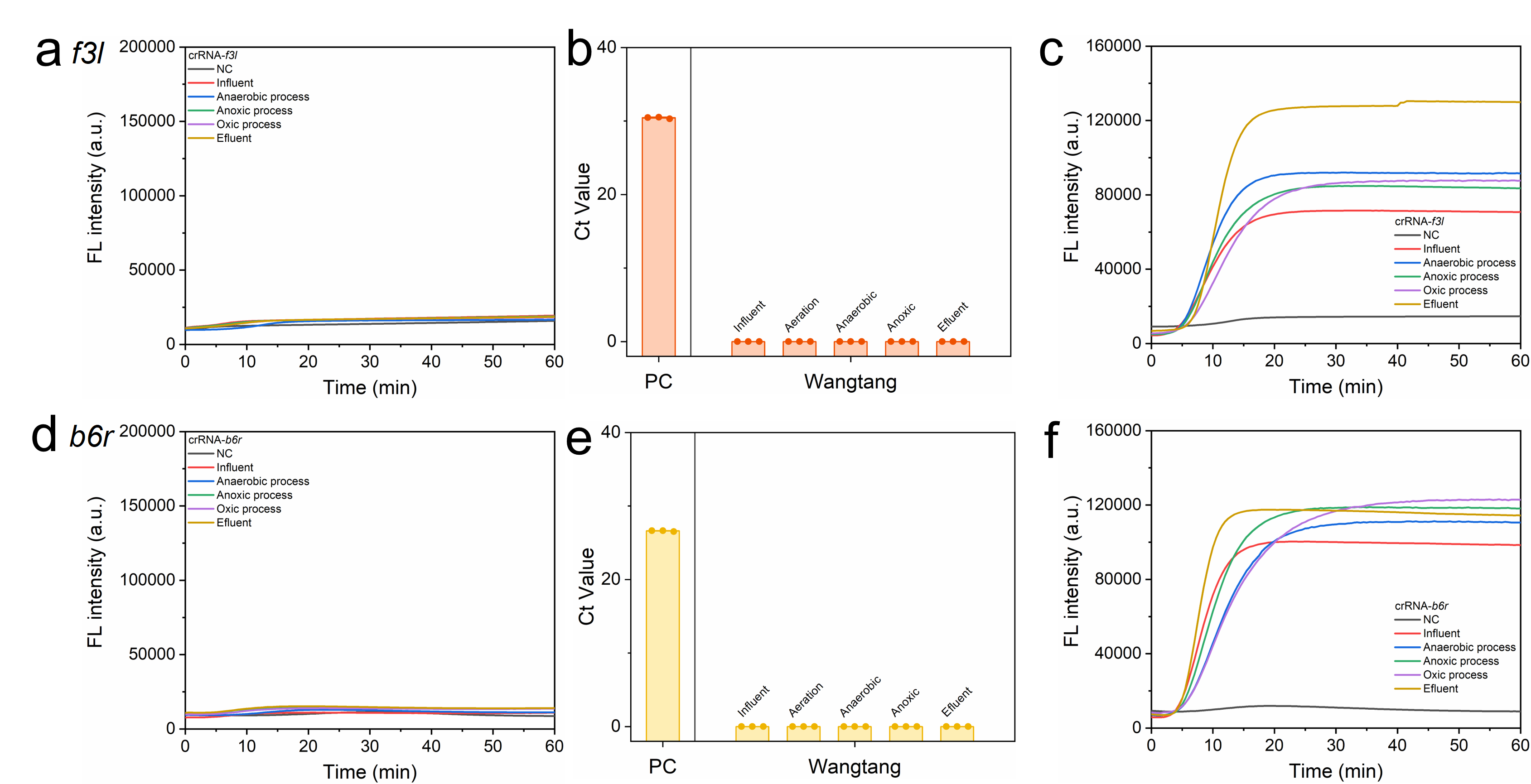


**Supplementary Fig. 16.** **Mpox detection in urban sewage of Wangtang WWTP by WATER NEWS.** The mpox in the five wastewater samples collected from the influent, anoxic tank, anaerobic tank, oxic tank and effluent were detecting using WATER NEWS (**a**,**d**,**c**,**f**) and qPCR (**b**,**e**).


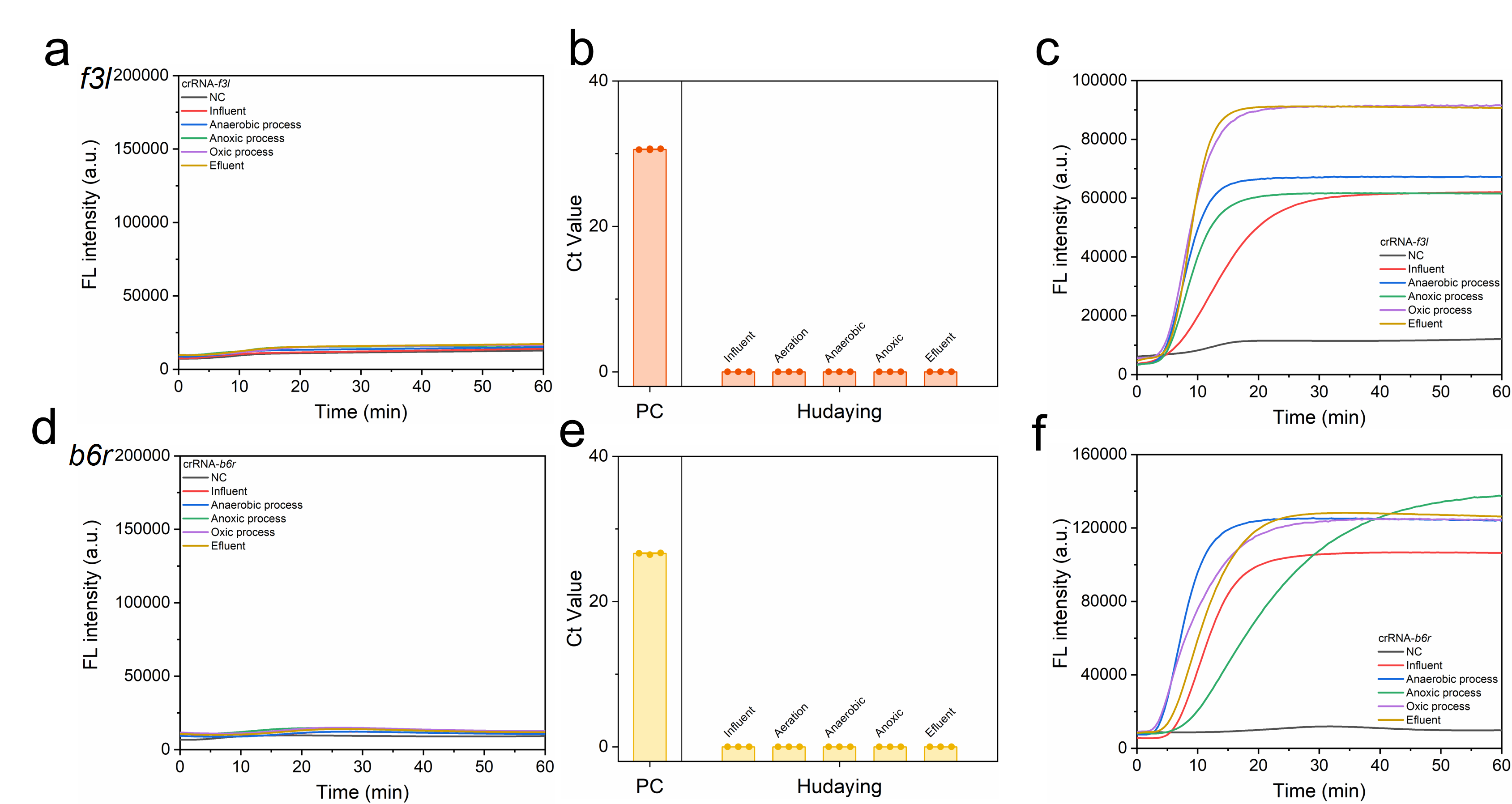


**Supplementary Fig. 17. Mpox detection in urban sewage of Hudaying WWTP by WATER NEWS.** mpox genome in the five wastewater samples collected from the influent, anoxic tank, anaerobic tank, oxic tank and effluent was detecting using WATER NEWS (**a**,**d**,**c**,**f**) and qPCR (**b**,**e**).


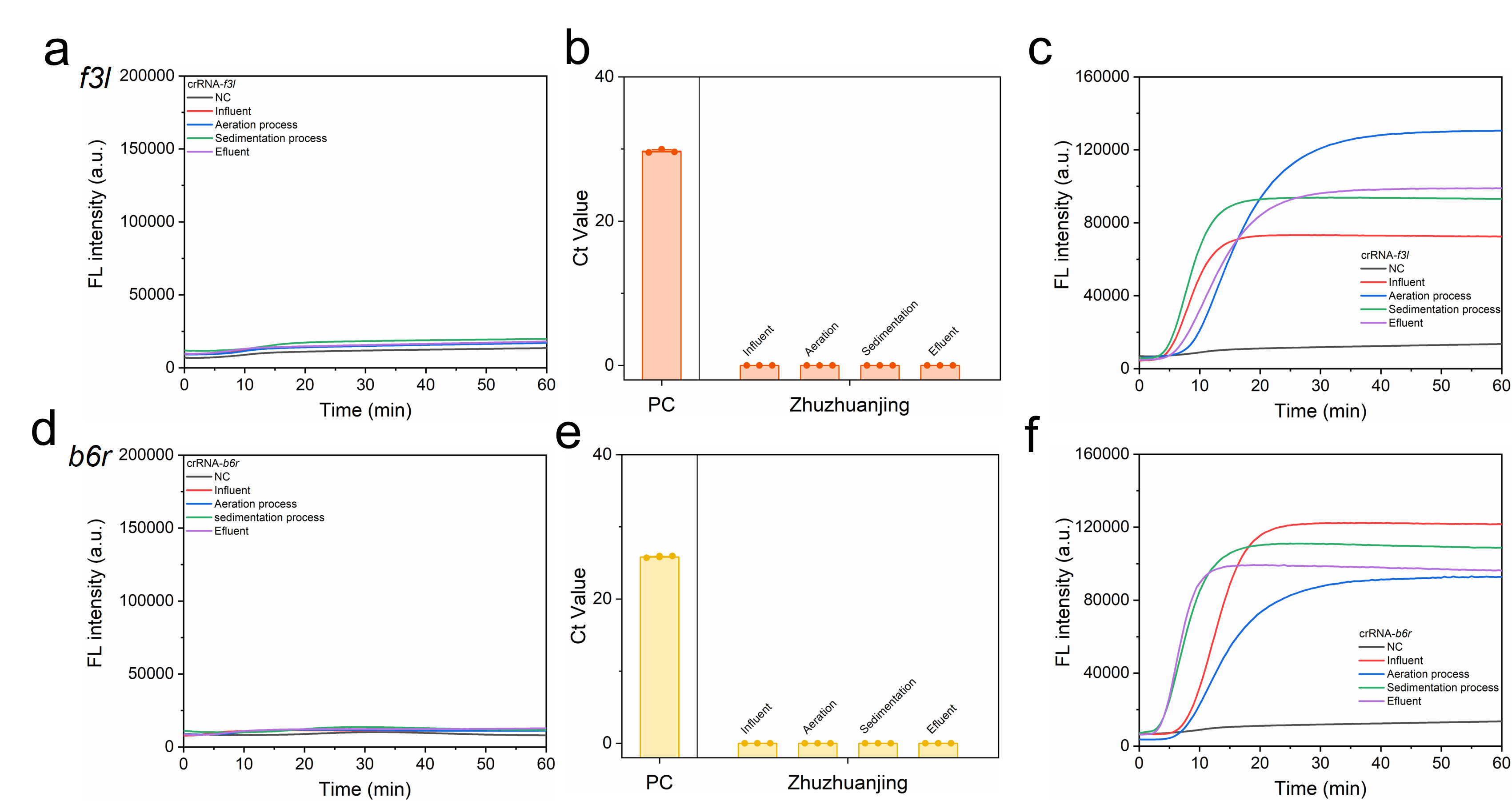


**Supplementary Fig. 18. Mpox detection in urban sewage of Zhuzhuanjing WWTP by WATER NEWS.** mpox genome in the four wastewater samples collected from the influent, aeration tank, sedimentation tank, and effluent was detecting using WATER NEWS (**a**,**d**,**c**,**f**) and qPCR (**b**,**e**).


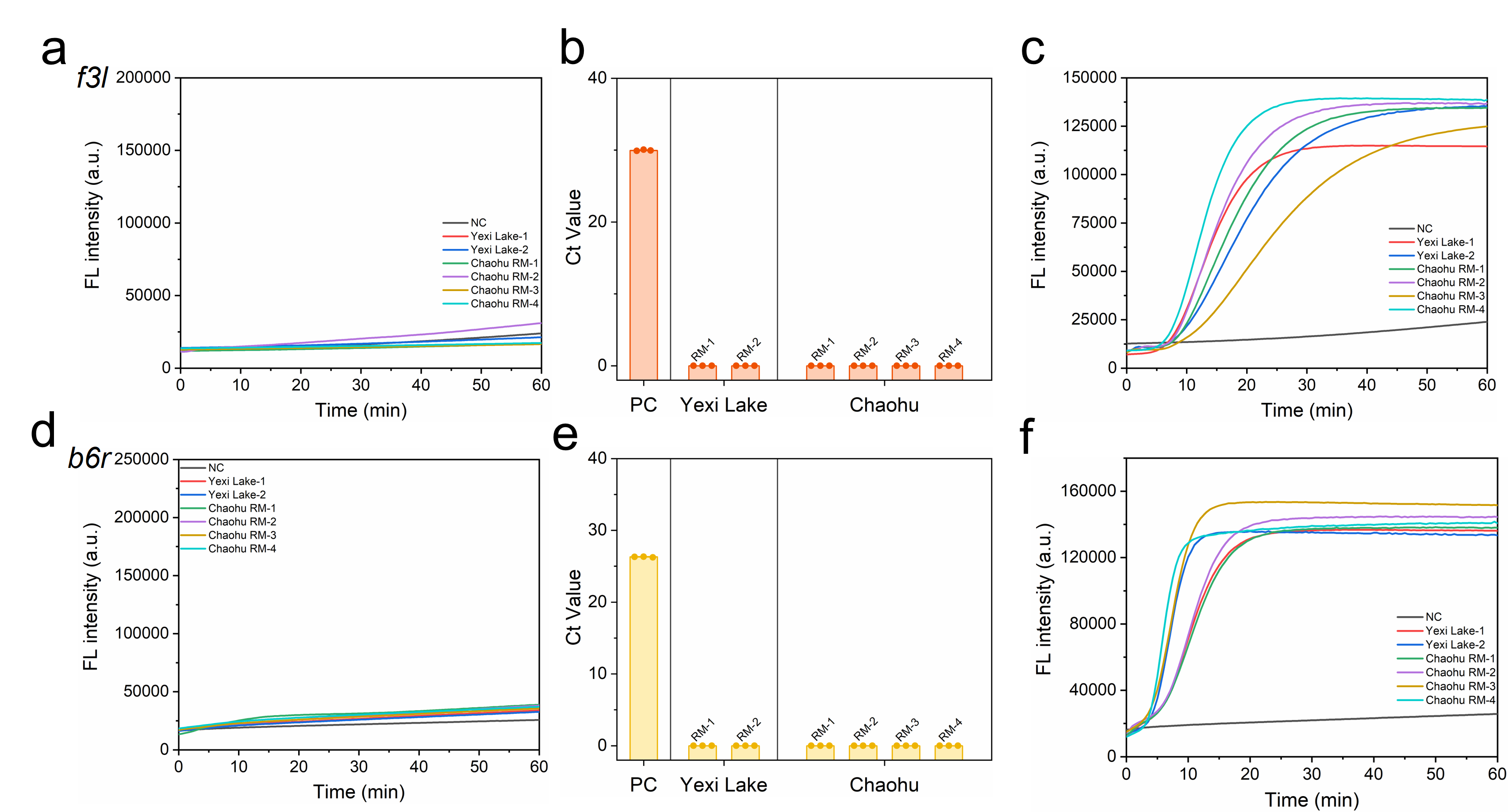


**Supplementary Fig. 19. Mpox detection in natural water samples by WATER NEWS.** mpox genome in the two samples from Yexi Lake and the four samples from Chaohu Lake entrance were detecting using WATER NEWS (**a**,**d**,**c**,**f**) and qPCR (**b**,**e**).


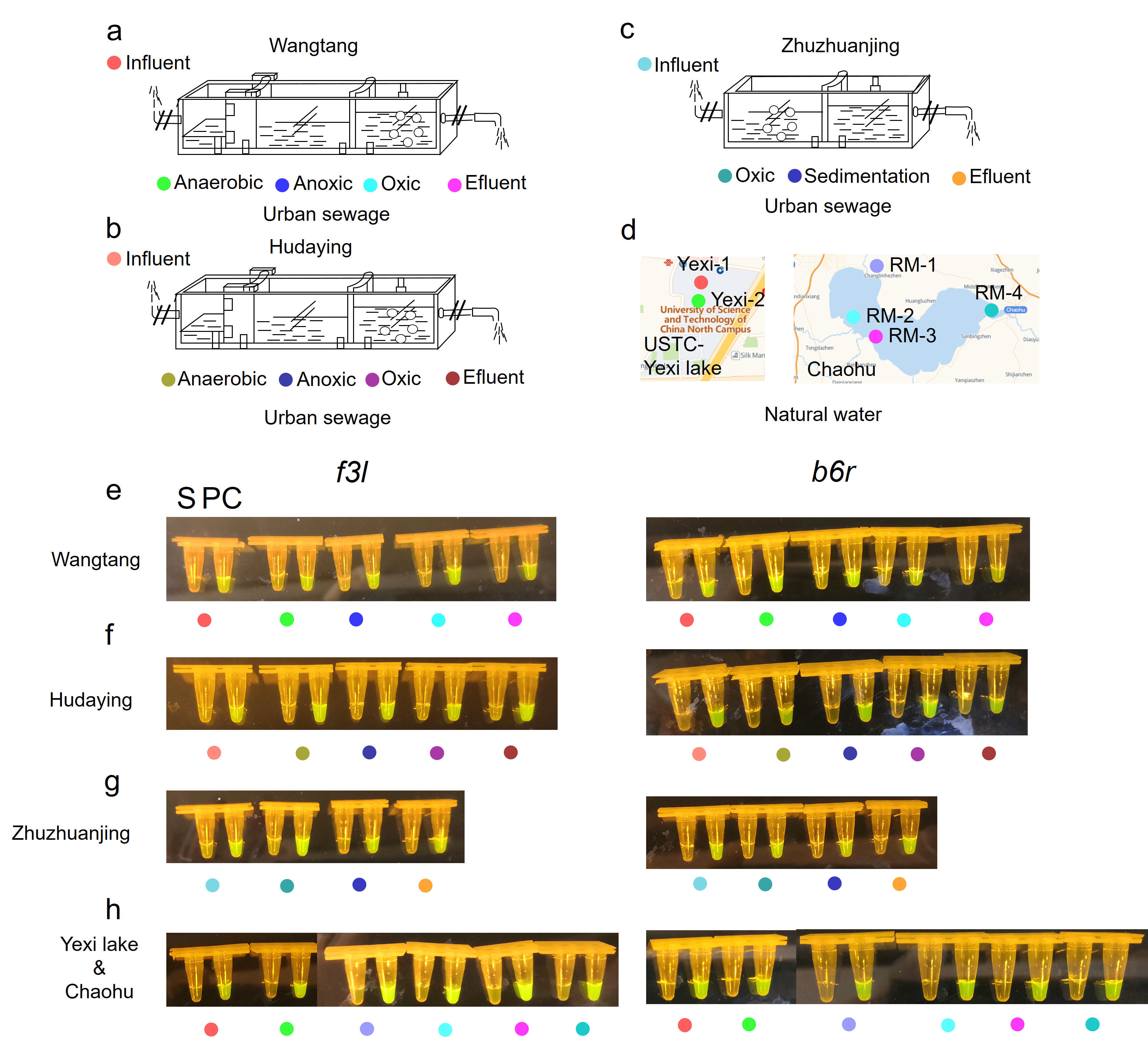


**Supplementary Fig. 20. Mpox detection in urban sewage and natural water based on in-tube** **fluorescence readout.** S stands for original samples, PC stands for the contrived samples.


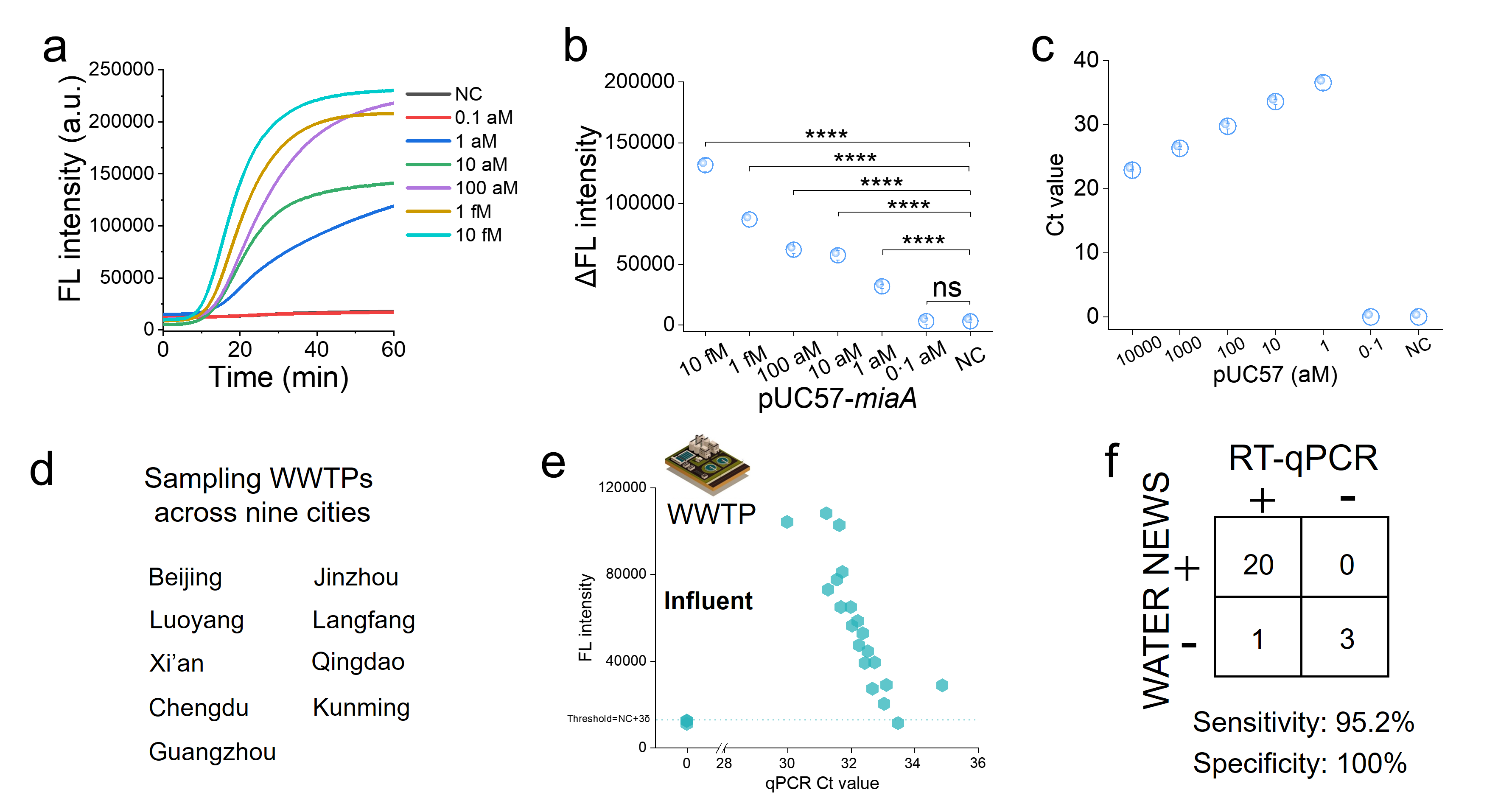


**Supplementary Fig. 21.** **Detection of *Pseudomonas aeruginosa* using WATER NEWS. a–b,** The LoD of the WATER NEWS assay was evaluated using the conserved *miaA* gene as the target. **c,** The LoD of qPCR targeting the same *miaA* gene was assessed for comparison. **d–e,** Influent samples from 24 WWTPs across China were analyzed for *P. aeruginosa* to evaluate the specificity and sensitivity of the WATER NEWS assay.


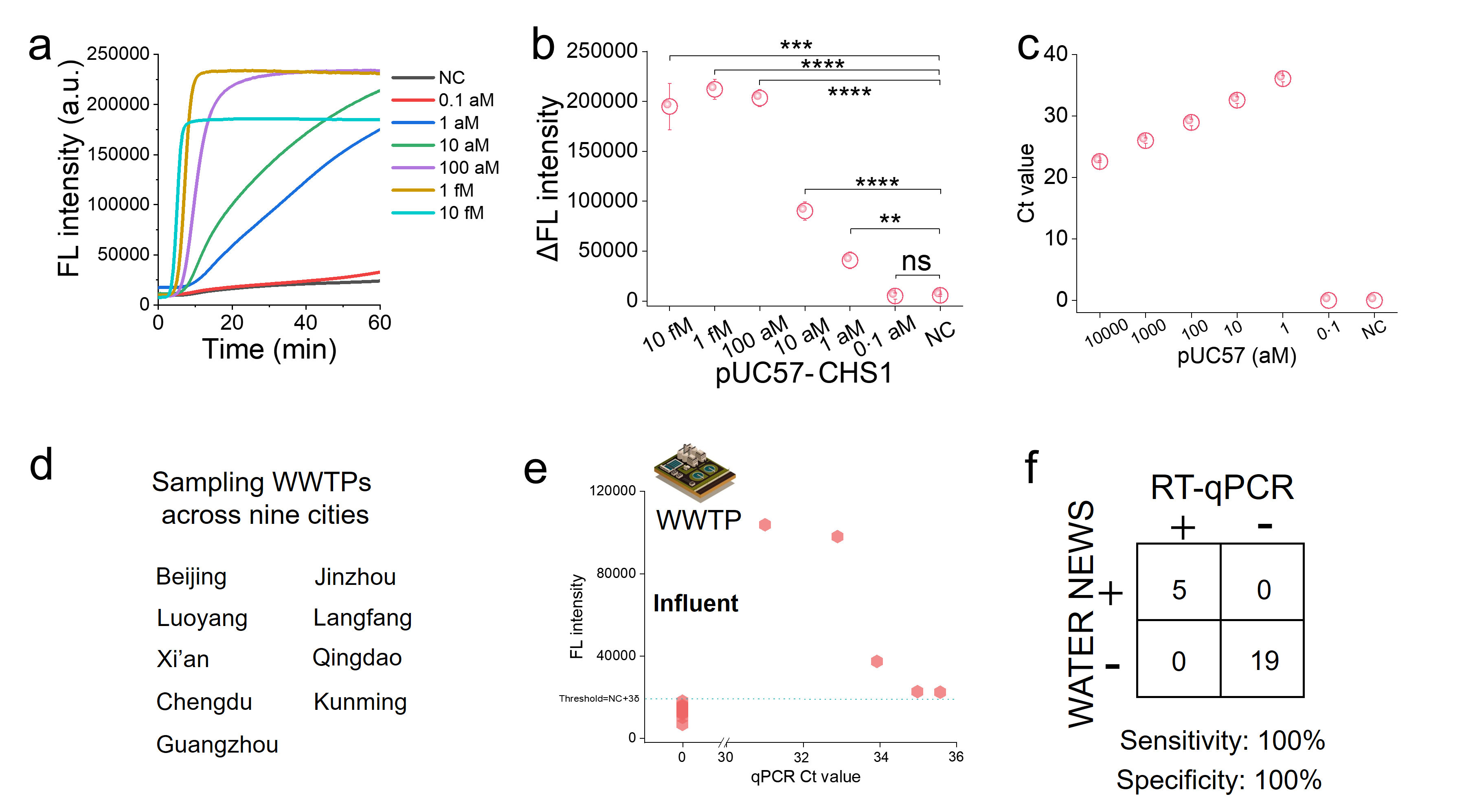


**Supplementary Fig. 22. Detection of *Candida albicans* using WATER NEWS. a–b,** The LoD of the WATER NEWS assay was evaluated using the *C. albicans* SC5314 CHS1 gene (Gene ID: 3641364) as the target. **c,** The LoD of qPCR targeting the same gene was assessed for comparison. **d–e,** Influent samples from 24 WWTPs across China were analyzed for *C. albicans* to evaluate the specificity and sensitivity of the WATER NEWS assay.

**Supplementary Fig. 23.** **Evaluation of specificity for the SARS-CoV-2 detection using WATER NEWS.** Using a one-pot detection system targeting the *N* gene, we tested the SARS-CoV-2, pseudoviruses with the *f3l* or *b6r* gene，*Helicobacter pylori*, *Pseudomonas aeruginosa* PAO1, *Aeromonas hydrophila* ATCC 7966, *Shewanella putrefaciens* CN32, and *Escherichia coli* MG1655.


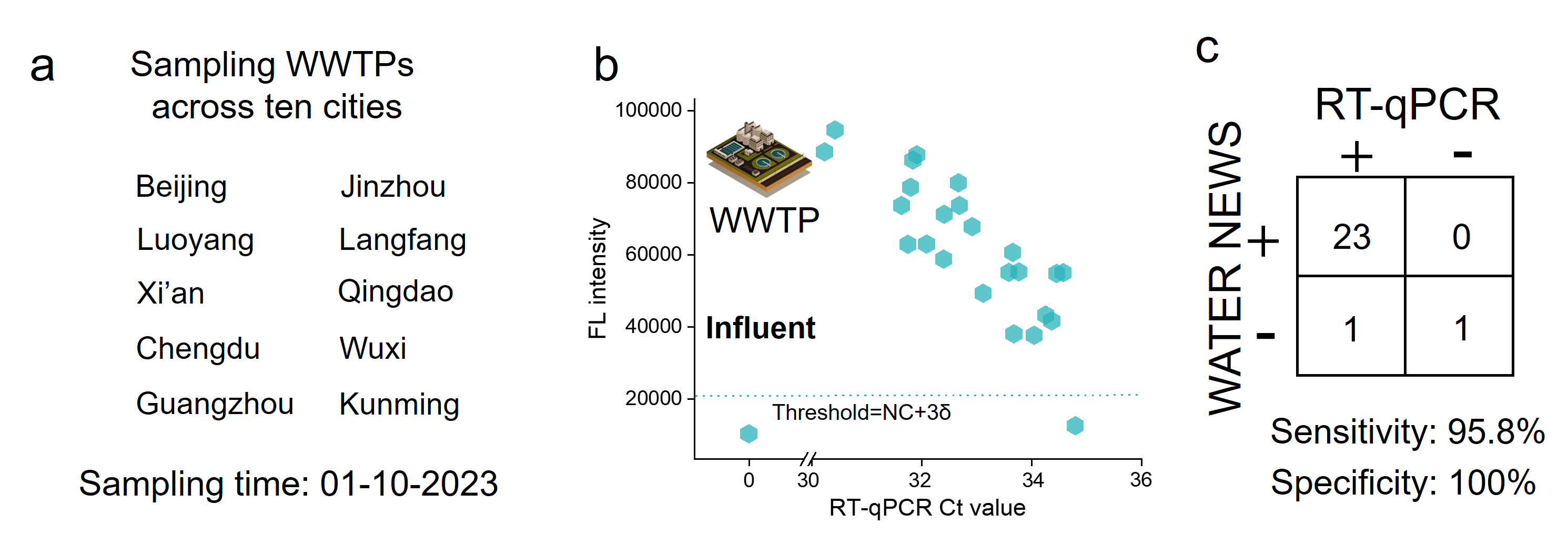


**Supplementary Fig. 24.** **SARS-CoV-2 detection in wastewater by WATER NEWS. a-c**, On January 10th, 2023, as the COVID-19 infection wave gradually declined, influent samples were collected from 25 WWTPs across 10 cities in China. The nucleic acids were extracted and stored at -80℃ for preservation, and subsequently these samples were tested using the WATER NEWS. The threshold was set as the mean of three negative experiments plus three times the standard deviation. If the fluorescence value exceeded this threshold, it was defined as positive; otherwise, it was defined as negative.

**References**

[1] Zheng X, Wang M, Deng Y, Xu X, Lin D, Zhang Y, et al. A rapid, high-throughput, and sensitive PEG-precipitation method for SARS-CoV-2 wastewater surveillance. *Water Research*. 2023; **230**: 119560.

[2] Ngwira LG, Sharma B, Shrestha KB, Dahal S, Tuladhar R, Manthalu G, et al. Cost of wastewater-based environmental surveillance for SARS-CoV-2: Evidence from pilot sites in Blantyre, Malawi and Kathmandu, Nepal. *PLOS global public health*. 2022;**2**:e0001377.

[3] Creese A PD. Cost analysis in primary health care : a training manual for programme managers: Geneva: World Health Organization 1994: https://iris.who.int/handle/10665/40030.

[4] Yoo B-K, Iwamoto R, Chung U, Sasaki T, Kitajima M. Economic Evaluation of Wastewater Surveillance Combined with Clinical COVID-19 Screening Tests, Japan. *Emerging Infectious Diseases*. 2023;**2 9**: 1608-1617.

[5] Bustin SA, Benes V, Garson JA, Hellemans J, Huggett J, Kubista M, et al. The MIQE Guidelines: Minimum Information for Publication of Quantitative Real-Time PCR Experiments. *Clinical Chemistry*. 2009; **55**: 611-622.

[6] Chinese Center for Disease Control and Prevention. The epidemic situation of SARS-CoV-2 infection in China 2024. https://en.chinacdc.cn/.

[7] Ng W-Y, Thoe W, Yang R, Cheung W-P, Chen C-K, To Kh. et al. The city-wide full-scale interactive application of sewage surveillance programme for assisting real-time COVID-19 pandemic control-A case study in Hong Kong. *Science of the Total Environment*. 2023; **875**：162661.

[8] Gootenberg JS, Abudayyeh OO, Lee JW, Essletzbichler P, Dy AJ, Joung J, et al. Nucleic acid detection with CRISPR-Cas13a/C2c2. *Science*. 2017; **356**: 438-442.
